# Isolaurenidificin and Bromlaurenidificin, Two New C_15_-Acetogenins from the Red Alga *Laurencia obtusa*

**DOI:** 10.3390/molecules22050807

**Published:** 2017-05-15

**Authors:** Nahed O. Bawakid, Walied M. Alarif, Najla A. Alburae, Hajer S. Alorfi, Khalid O. Al-Footy, Sultan S. Al-Lihaibi, Mohamed A. Ghandourah

**Affiliations:** 1 Department of Chemistry, Faculty of Science, King Abdulaziz University, P.O. Box 80203, Jeddah 21589, Saudi Arabia; nbawaked@kau.edu.sa (N.O.B.); halaorfi@kau.edu.sa (H.S.A.); Kalfooti@kau.edu.sa (K.O.A.-F.); 2 Department of Marine Chemistry, Faculty of Marine Sciences, King Abdulaziz University, P.O. Box 80207, Jeddah 21589, Saudi Arabia; sallihaibi@kau.edu.sa (S.S.A.-L.); mghandourah@kau.edu.sa (M.A.G.); 3 Department of Biology, Faculty of Sciences, King Abdulaziz University, P.O. Box 80207, Jeddah 21589, Saudi Arabia; nalbourae@gmail.com

**Keywords:** marine algae, fatty acids, halogenations, spectroscopy, anti-inflammatory

## Abstract

Chromatographic fractionation of the CH_2_Cl_2_/MeOH extract of the Red Sea red alga *Laurencia obtusa* gave two new hexahydrofuro[3,2-*b*]furan-based C_15_-acetogenins, namely, isolaurenidificin (**1**) and bromlaurenidificin (**2**). The chemical structures were elucidated based on extensive analyses of their spectral data. Compounds **1** and **2** showed no toxicity (*LC*_50_ > 12 mM) using *Artemia salina* as test organism. Both compounds showed weak cytotoxicity against A549, HepG-2, HCT116, MCF-7, and PC-3 cells, however, they exhibited a relatively potent cytotoxic activity against peripheral blood neutrophils. This can be attributed partly to induction of apoptosis.

## 1. Introduction

Red algae of Genus *Laurencia* (Division Rhodophyta, Order Ceramiales, Family Rhodomelaceae) are one of the most studied marine organisms [1]. They are found on the inter-tidal rocks of the warm sea waters throughout the world [2]. There are 421 species (and infra specific) names in the corresponding database at present, of which 146 have been flagged as currently accepted taxonomically [3]. It has been proved that the genus *Laurencia* is a rich source of structurally unique secondary metabolites, characterized by a relatively high degree of halogenation and core terpene structures [4]. It is believed that these chemical compounds serve as a defense mechanism [5]. The halogenated metabolites from *Laurencia* possess several biological activities, such as antifeedant, anthelmintic, antimalarial, antifouling, antimicrobial and cytotoxicity [6,7,8].

C_15_-acetogenins are fatty acid derived compounds with a conjugated enyne or allene terminus. The presence of one or more halogen atom (usually a bromine atom) and uncommon cyclic ethers with different ring sizes are the major features of the algal acetogenins [9]. C_15_-acetogenins are exclusive to the red algae of the genus *Laurencia* and some of their herbivores as well [10]. In continuation with our program aiming at isolation of new chemical structures and/or biological active secondary metabolites from marine algae [11,12,13,14]; *L*. *obtusa* was collected in May 2016 from Salman Gulf, near Jeddah, dried and extracted. The methylene chloride (CH_2_Cl_2_) soluble fraction was sequentially partitioned on Al oxide and Sephadex LH-20 columns. Promising fractions were further purified using preparative TLC.

The present study was designed to isolate and characterize novel metabolites from *Laurencia obtusa*, and to evaluate their potential cytotoxic activity against *Artemia salina* and selected human cancer cells (A549 lung carcinoma cells, HepG2 hepatocellular carcinoma cells, HCT116 colorectal carcinoma cells, MCF-7 breast cancer cells and PC-3 prostate cancer cells).

## 2. Results and Discussion

**Figure 1:**
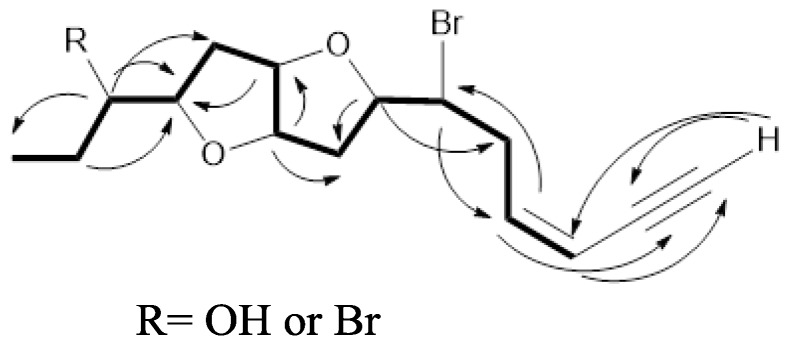
Selected COSY (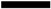) and HMBC (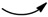) correlations of **1** and **2**.

**Figure 2:**
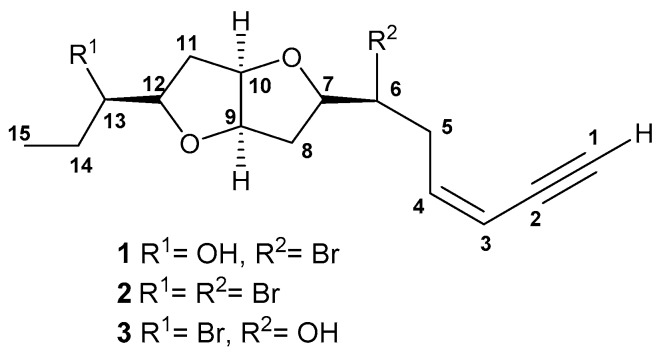
Chemical structures of compounds **1**–**3**.

Compound **1** was isolated as a colorless oil, ${\left[\alpha \right]}_{D}^{22}$= −18.0 (*c* 0.016, CH_2_Cl_2_). The molecular formula was determined as C_15_H_21_BrO_3_, employing positive mode HR-ESI-MS, requiring five degrees of unsaturation. EIMS showed a characteristic molecular-ion cluster at *m*/*z* 328 and 330 in 1:1 ratio, which clearly indicated the presence of one Br atom. The presence of terminal acetylenic and hydroxyl groups and a furan ring were evidenced from the infrared spectroscopy (IR) absorptions at υ_max_3292, 3481and 1252, and 1080 cm^−1^, respectively. The ultraviolet (UV) spectrum exhibited the presence of a conjugated enyne function absorbing at 223 nm. The ^13^C-NMR spectrum displayed 15 signals, categorized by a distortionless enhancement by polarization transfer (DEPT) experiment into one methyl, four methylene, ninemethine, and one quaternary carbons (Figure S1f–S1h. ^1^H and heteronuclear single quantum coherence (HSQC) NMR spectra led to the assignment of the following features: one acetylenic proton resonating at δ_H_/δ_C_ 3.15 (d, *J* = 1.7 Hz)/82.8; two olefinic methine protons 6.18 (ddd, *J* = 11.1, 10.2, 7.7 Hz)/141.1 and 5.63 (ddd, *J* = 11.1, 3.4, 1.7 Hz)/111.0; one hydroxylated methine 3.54 (ddd, *J* = 11.1, 6.8, 4.3 Hz)/75.2; one halogenated methine 4.15 (ddd, *J* = 10.2, 8.5, 4.3 Hz)/55.7; four etheric methine protons 4.55 (ddd, *J* = 6.0, 4.3, 1.7 Hz)/84.3, 4.53 (ddd, *J* = 6.0, 4.3, 1.7 Hz)/85.0, 4.08 (dd, *J* = 14.5, 7.7 Hz)/83.6, and 3.88 (ddd, *J* = 14.7, 8.5, 6.8 Hz)/84.3, four methylenes assigned to carbons resonating at δ_C_ 36.8, 35.5, 34.9, and 26.5; and a tertiary methyl at δ_H_/δ_C_ 1.00 (t, *J* = 6.8 Hz)/10.1 carbons (Figures S1a–S1e and S1l–S1n). Interpretation of the ^1^H-^1^H correlation spectroscopy (COSY) spectrum revealed the presence of one large spin sequence system from H-3 to H-15, as well as a long rang coupling of H-1 with H-3 (Figure 1), (Figure S1i–S1k). The presence of terminal conjugated enyne was proposed from a HMBC correlation from H-3 at δ_H_ 5.63 to C-1 (CH, 82.8), C-2 (C, 79.9) and C-4 (CH, 141.1). The presence of a OH function at C-12 was concluded from the HMBC correlation between both the Me protons at δ_H_ 1.0 (H-15) and the ether proton at 3.88 (H-12) with C-13 (CH, 75.2) (Figure S1i–S1k). The relative configuration of **1** was determined by a combination of data from the nuclear Overhauser effect spectroscopy (NOESY) spectrum and the coupling constant values (*J*). NOESY enhancements were observed between H-9 (δ_H_ 4.55) and H-10 (δ_H_ 4.53), H-7 (δ_H_ 4.08) and H-12 (δ_H_ 3.88) (Figure S1r), suggesting their co-facial orientation, Extensive comparison between the spectral data of **1** with those reported for laurenidificin (**3**, Figure 2) isolated from *L*. *nidifica* [15], revealed the similarity in gross carbon skeleton and orientation of Hα-9 (owing to the similar *J* values),with the difference of the switched positions of the Br atom and OH function. The trivial name isolaurenidificin was given to compound **1** (Figure 2).

Compound **2** was isolated as colorless oil, ${\left[\alpha \right]}_{D}^{22}$ = − 20.5 (c 0.005, CH_2_Cl_2_). The molecular formula was determined as C_15_H_20_Br_2_O_2_ employing positive mode HR-ESI-MS, requiring five degrees of unsaturation. EIMS showed a characteristic molecular-ion cluster at *m*/*z* 389, 391 and 393 in 1:2:1 ratio, which clearly indicated the presence of two Br atoms. The presence of a terminal acetylenic group and a furan ring were evidenced from the IR absorptions at υ_max_3294, 1236 and 1070 cm^−1^, respectively. The UV spectrum exhibited the presence of a conjugated enyne function absorbing at 222 nm. The ^13^C-NMR spectrum displayed 15 signals, categorized by DEPT experiment into one methyl, four methylene, nine methine, and one quaternary carbons (Figure S2f–S2h). ^1^H- and HSQC-NMR spectra led to the assignment of the following features:one acetylenic proton resonating at δ_H_/δ_C_ 2.76 (d, *J* = 1.7 Hz)/82.8; two olefinic methine protons 5.85 (ddd, *J* = 11.9, 11.1, 6.8 Hz)/141.4 and 5.35 (ddd, *J* = 11.1, 2.6, 1.7 Hz)/110.6; two halogenated methines4.24 (ddd, *J* = 11.9, 9.4, 2.6 Hz)/61.4 and 3.83 (m)/55.0; four etheric methine protons 3.99 (ddd, *J* = 6.0, 3.4, 1.7 Hz)/84.9, 3.93 (ddd, *J* = 9.4, 8.5, 6.0 Hz)/83.6, 3.82 (m)/84.1, and 3.56 (ddd, *J* = 12.8, 7.7, 5.1 Hz)/82.7, four methylenes assigned to carbons resonating at δ_C_ 37.5, 36.9, 35.4, and 28.4; and a tertiary methyl at δ_H_/δ_C_ 1.04 (t, *J* = 7.7 Hz)/11.2 carbons (Figures S2a–S2e and S2l–S2m). After examination of the^1^H-^1^HCOSY and HMBC correlation spectra (Figure 1), **2** is very similar to **1** with the exception of the appearance of halogenated methine proton signal in **2** instead of a hydroxyl methine proton signal in **1**. The relative configuration of **2** was determined by the combination of both data from NOESY spectrum and the coupling constant values (*J*). Compound **2** was found also to be similar to **1**, where signals due to H-7, H-9, H-10 and H-12 are correlated together, implying a co-facial orientation of these protons (Figure S2o–S2p). The trivial name brom laurenidificin was given to **2** (Figure 2).

**Figure 3:**
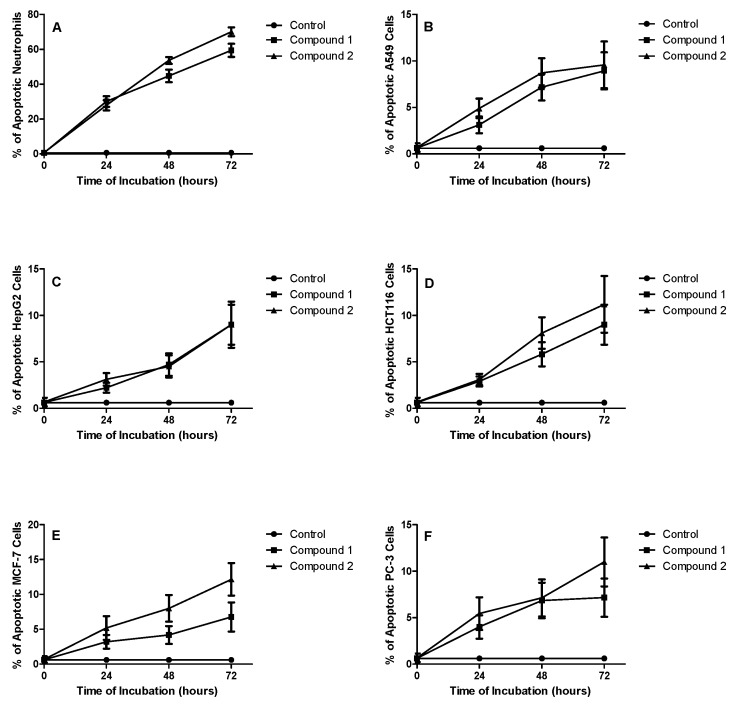
Induction of apoptosis by compounds **1** and **2** in peripheral blood neutrophils (**A**); A549 lung carcinoma cells (**B**); HepG2 hepatocellular carcinoma cells (**C**); HCT116 colorectal carcinoma cells (**D**); MCF-7 Breast cancer cells (**E**) and PC-3 prostate cancer cells (**F**). Each compound was used at a concentration of 50 mM/L.

As aforementioned the genus *Laurencia* generates unusual secondary metabolites. The vast majority of these compounds are halogenated diterpenes, sesquiterpenes and C_15_ non-terpenoids containing different functions, including acetylenic, vinyl acetylenic or allenic side chains. The diversity of their molecular structures enables them to exhibit different bioactivities [11,12,13,14]. Therefore, compounds **1** and **2** were evaluated against *Artemia salina.* The assay is a rapid and inexpensive screen for potential cytotoxicity [16]. This assay gave a preliminary overview of the cytotoxic activity of the tested compounds, and the toxicity of **1** and **2** was then further investigated in a dose-response study determine the *IC*_50_ values of compounds **1** and **2** against each cell type after a 24 h incubation period. The obtained *IC*_50_ of each cell line was then used to establish the time-course study of the apoptotic activity as shown in Figure 3. In general, cytotoxicity of **1** and **2** againstA549 lung carcinoma cells, HepG2 hepatocellular carcinoma cells, HCT116 colorectal carcinoma cells, MCF-7 breast cancer cells and PC-3 prostate cancer cells was weak, as indicated by the high *IC*_50_ values (>15 mM, Table 1). However, the *IC*_50_s of the tested compounds at 24 h of incubation was 14 µM for **1** and 11 µM for **2** versus dexamethasone 0.9 µM. These observations were further substantiated by assessing their effects on cellular apoptosis. Degradation of DNA into a specific fragmentation pattern is a characteristic feature of apoptosis. In contrast to the random fragmentation with necrosis, apoptosis-associated DNA fragmentation is characterized by cleavage of the DNA at regular intervals, visualized on agarose gel electrophoresis as a DNA ladder consisting of multimers of approximately 200 base pairs. Blood neutrophils as well as the five cell lines were prepared, cultured, and incubated for 24, 48 and 72 h in medium with and without the isolated compounds (Table 2). Both morphology and DNA fragmentation methods assessed the percentage of neutrophils apoptosis in each culture. The data in Figure 3 indicate a relatively potent apoptosis-inducing activity of both compounds **1** and **2** against peripheral blood neutrophils. However, the fraction of apoptotic tumor cells exposed to both compounds never exceeded 15% even at 72 h of incubation. It is well documented that cell death embraces apoptosis, necrosis, autophagy and others [17]. Thus, it can be concluded that other types of cell death cannot be excluded.

## 3. Experimental

### 3.1. General

Silica gel GF 254 (Merck, Darmstadt, Germany) was used for analytical thin layer chromatography (TLC). Preparative thin layer chromatography (PTLC) was performed on aluminum oxide plates (20 × 20 cm) of 250 µm thickness. Plates were sprayed with *p*-anisaldehyde-sulphuric acid reagent and heated at 100 °C for 1–2 min. for detection. Electron impact mass spectra were determined at 70 ev on a Kratos (Manchester, UK) MS-25 instrument. 1D and 2D NMR spectra were recorded by using a Bruker (Karlsruhe, Germany) AVANCE III WM 850 MHz spectrometer and ^13^C-NMR spectra were recorded at 212.5 MHz. Tetramethylsilane (TMS) was used as internal standard. The deuterated solvents deuterated chloroform (CDCl_3_) and hexadeuterated benzene (C_6_D_6_) were purchased from Sigma-Aldrich (Steinheim, Germany). Sulforhodamine-B was purchased from Sigma-Aldrich (St. Louis, MO, USA). RPMI-1640 medium, heat inactivated fetal bovine serum, streptomycin, penicillin and other cell culture materials were purchased from Invitrogen (Carlsbad, CA, USA). Other reagents were of the highest analytical grade.

### 3.2. Extraction and Isolation

*Laurencia obtusa* was collected in May 2016 from Salman Gulf, north of Jeddah, Saudi Arabia. The voucher sample (JAD 03060) was deposited at the Marine Chemistry Department, King Abdulaziz University, Jeddah, Saudi Arabia. It was dried then extracted with equal volumes of CH_2_Cl_2_ (dichloromethane)/MeOH (methanol). The residue (6 g) was subjected to column chromatography on aluminum oxide using gradient elution with *n*-hexane/diethyl ether (0–50% diethyl ether), followed by then *n*-hexane/ethyl acetate (10–50% ethyl acetate). Fractions of 25 mL were gathered and monitored using TLC. The promising fractions were further purified by PTLC and Sephadex LH-20 chromatography.

### 3.3. Spectral Data

*Isolaurenidificin* (**1**). The fraction eluted with 30% ethyl acetate was purified by preparative TLC system using *n*-hexane/ethylacetate (7:3). The green color zone with *p*-anisaldehyde-sulfuric acid was collected to provide 2.7 mg of colorless oil; *R*_f_ 0.20; ${\left[\alpha \right]}_{D}^{22}$ = −18.0 (*c* 0.016, CH_2_Cl_2_); UV (MeOH) λ_max_ 223 nm; IR υ_max_ 3481, 3292, 2924, 2854, 1729, 1663, 1461, 1252 and 1080 cm^−1^; electrospray ionization-high resolution mass spectrometry (ESI-HRMS) *m*/*z* 351.0559, 353.0538 [M + Na]^+^ (1:1) (calcd. for C_15_H_21_^79^BrO_3_Na, 351.0572; C_15_H_20_^81^BrO_3_Na, 351.0551; respectively); ^1^H-, ^13^C-NMR (CDCl_3_) δ ppm (Table 3).

*Bromlaurenidificin* (**2**). The fraction eluted with 15% diethyl ether was purified by preparative TLC system using *n*-hexane/diethyl ether (8.5:1.5). The greenish-gray color zone with *p*-anisaldehyde-sulfuric acid was collected to provide 1.1 mg of colorless oil; *R*_f_ 0.20; ${\left[\alpha \right]}_{D}^{22}$ = −20.5 (*c* 0.005, CH_2_Cl_2_); UV (MeOH) λ_max_ 222 nm; IR ν_max_ 3294, 2923, 2853, 1736, 1461, 1378, 1236 and 1070 cm^−1^; ESI-HRMS *m*/*z* 412.9728, 414.9707, 416.9687 [M + Na]^+^ (1:2:1) (calcd. for C_15_H_20_^79^Br_2_O_2_Na, 412.9716; C_15_H_20_^79^Br^81^BrO_2_Na, 414.9695; C_15_H_20_^81^Br_2_O_2_Na, 416.9673; respectively); ^1^H-, ^13^C-NMR (C_6_D_6_) δ ppm (Table 3).

### 3.4. Toxicity of the Isolated Compounds

#### 3.4.1. Brine Shrimp Lethality (*Artemia salina*) Assay

A solution of sea water was made by dissolving 32.5 g (a natural blend of salts and trace element for sea water fish (Sera Company, AquaristikGmbh, Henisberg, Germany) in distilled water. Brine shrimp *Artemia salina* (Leach), eggs (ca. 1 mg) were placed in a hatching chamber (22 × 32 cm). The hatching chamber was kept under an inflorescent bulb for 48 h for the eggs to hatch into shrimp larvae (nauplii). One mg of each pure compound was dissolved in 5 mL of solvent in which it was soluble and from this stock solution was transferred to vials corresponding to (0.01 to 100 mM), respectively. Each dosage was tested in triplicate. Ten larvae (nauplii) of *Artemia salina* were transferred into each vial and the volume made into 5 mL with sea salt solution, immediately after adding the nauplii, 24 h later, the number of surviving shrimp at each dosage was counted B recorded. *LC*_50_ values were determined statistically [16].

#### 3.4.2. Cytotoxicity Bioassay

##### Cell Culture

The culture medium for A549 lung carcinoma cells and PC-3 prostate cancer cells were Dulbecco’s Modified Eagle’s Medium (DMEM) formulated with high glucose, while Eagle Minimum Essential Medium (EMEM) was used for HepG2 hepatocellular carcinoma cells and MCF-7 Breast cancer cells. Finally, McCoy’s 5A medium, include 2 mM glutamine + 10% fetal bovine serum (FBS), was employed for HCT116 colorectal carcinoma cells. The cell lines were obtained from the National Cancer Institute (Cairo, Egypt) and maintained in Roswell Memorial Institute (RPMI)-1640 medium supplemented with 100 µg/mL of streptomycin, 100 units/ mL of penicillin and 10% of heat-inactivated fetal bovine serum (Invitrogen) in a humidified atmosphere containing 5% (*v*/*v*) CO_2_ at 37 °C.

##### Cytotoxicity Assay

Compounds **1** and **2** were tested against peripheral blood neutrophils, A549 lung carcinoma cells, HepG2 hepatocellular carcinoma cells, HCT116 colorectal carcinoma cells, MCF-7 breast cancer cells and PC-3 prostate cancer cells. The percentage of viability of cell was estimated by using doxorubicin as a positive standard anticancer drug. These assays had been performed according to the published protocols [18]. The final concentration of dimethylsulphoxide (DMSO) in each sample did not exceed 0.1% *v*/*v*. The cancer cells were batch cultured for 10 d, then seeded in 96 well plates of 10 × 10^3^ cells/well in fresh complete growth medium in 96-well microtiter plastic plates at 37 °C for 24 h under 5% CO_2_ using a water jacketed carbon dioxide incubator. The medium was added and cells were incubated either alone (negative control) or with different concentrations of sample to give a range of concentrations (0.01 to 100 mM). Each cell type was suspended in the suitable medium, 1% antibiotic-antimycotic mixture (104 µg/mL potassium penicillin, 104 µg/mL streptomycin sulfate and 25 µg/mL amphotericin B and 1% l-glutamine in 96-well flat bottom micro-plates at 37 °C under 5% CO_2_. After 96 h of incubation, the medium was again aspirated, trays were inverted onto a pad of paper towels, the remaining cells rinsed carefully with medium, and fixed with 3.7% (*v*/*v*) formaldehyde in saline for at least 20 min. The fixed cells were rinsed with water, and examined. The cytotoxic activity was identified as confluent, relatively unaltered mono-layers of stained cells treated with compounds. The *IC*_50_ was calculated based on the 50% loss of monolayer.

##### Apoptotic Effect on Neutrophils

Preparation of Blood Neutrophils: Neutrophils (>98% pure by May-Giemsa staining) were isolated from peripheral blood of normal healthy volunteer donors by a combination of dextran sedimentation and centrifugation through discontinuous plasma percoll gradients [19].

##### Culture of Neutrophils

Neutrophils were resuspended in an appropriate volume of RPMI 1640 medium with 10% autologous PRPDS and 100 μg/L of penicillin and streptomycin and divided into five equal volumes each put in culture tube. Cells were incubated (at 37 °C in a 5% carbon dioxide) as follows: (1) Only cells; (2) cells + DMSO at 0.01% *v*/*v*; (3) cells + each compound in DMSO at concentration of 50 mM/mL culture. The age of neutrophils in culture was calculated at the start of culture (zero time or base line), 24, 48, and 72 h [19]. 

##### Assessment of Cell Viability

At time 0 and then at subsequent times, cells were removed from culture and counted on a haemocytometer. Cell viability was determined by a Trypan Blue dye exclusion test; one volume of Trypan Blue (0.4% GiBCo, USA) was added to 5 volumes of cells at room temperature for 5 min. The *IC*_50_ values of isolated compounds were determined in comparison to dexamethasone [12,19].

## 4. Conclusions

This work reported two new hexahydrofuro[3,2-*b*]furan-based C_15_-acetogenins from an extract of the Red Sea red alga *Laurencia obtusa*. Both compounds showed weak cytotoxicity against A549, HepG-2, HCT116, MCF-7, and PC-3 cells. However, they exhibited a relatively potent cytotoxic effect against peripheral blood neutrophils with an *IC*_50_ of 14 µM for **1**, and 11 µM for **2** versus 0.9 µM for dexamethasone at 24 h of exposure. This can be attributed partly to induction of apoptosis.

## Figures and Tables

**Table 1:** The cytotoxic activity (*IC*_50_) values of **1** and **2** against human cancer cells. *

Compound	*IC*_50_ (mM)				
	A 549	HepG2	HCT116	MCF-7	PC-3
**1**	15.6	15.1	>20	15.5	17.9
**2**	15.9	15.3	17.9	17.9	>20

* A549 (lung carcinoma cells); HepG2 (hepatocellular carcinoma cells; HCT116 (colorectal carcinoma cells); MCF-7 (Breast cancer cells) and PC-3 (prostate cancer cells). All cells were incubated with compounds **1** or **2** for 72 h.

**Table 2:** Effect of the isolated compounds on apoptosis of peripheral blood neutrophils. *

Compound	% Apoptotic Neutrophils (Mean ± S.D.)			
	0 h	24 h	48 h	72 h
(Control)	0.41 ± 0.01	0.41 ± 0.01	0.41 ± 0.01	0.41 ± 0.01
**1**	0.63 ± 0.55	30.10 ± 3.11	44.86 ± 3.59	59.45 ± 3.76
**2**	0.80 ± 0.56	27.91 ± 2.93	53.62 ± 2.01	70.13 ± 2.51

* Neutrophils were incubated with compounds **1** or **2** for the assigned time.

**Table 3:** ^1^H- and ^13^C-NMR spectral data for compounds **1** ^a^ and **2** ^b^.

Position		1			2	
	δ_H_	Mult. *J* in Hz	δ_C_	δ_H_	Mult. *J* in Hz	δ_C_
1	3.15	d, *J =* 1.7	82.8	2.76	d, *J =* 1.7	82.8
2	-		79.9	-		79.8
3	5.63	ddd, *J =* 11.1, 3.4,1.7	111	5.35	ddd, *J =* 11.1, 2.6, 1.7	110.6
4	6.18	ddd, *J =* 11.1, 10.2, 7.7	141.1	5.85	ddd, *J =* 11.9, 11.1, 6.8	141.4
5	2.9	m	35.5	2.83	m	35.4
	2.96	dddd, *J =* 15.3, 8.5, 7.0, 1.7		2.87	m	
6	4.15	ddd, *J =* 10.2, 8.5, 4.3	55.7	3.83	m	55
7	4.08	dd, *J =* 14.5, 7.7	83.6	3.56	ddd, *J =* 12.8, 7.7,5.1	82.7
8	2.02	m	36.8	1.66	ddd, *J =* 13.6, 6.8, 1.7	36.9
	2.32	ddd, *J =* 13.6, 7.7, 6.8		1.85	ddd, *J =* 13.6, 6.0, 1.7	
9	4.55	ddd, *J =* 6.0, 4.3, 1.7	84.3	3.99	ddd, *J =* 6.0, 3.4,1.7	84.9
10	4.53	ddd, *J =* 6.0, 4.3, 1.7	85	3.82	m	84.1
11	2.02	m	34.9	1.91	ddd, *J =* 13.6, 7.7, 6.0	37.5
	2.22	ddd, *J =* 13.6, 7.7, 6.8		2.28	dd, *J =* 13.6, 5.1	
12	3.88	ddd, *J =* 14.7, 8.5, 6.8	84.3	3.93	ddd, *J =* 9.4, 8.5, 6.0	83.6
13	3.54	ddd, *J =* 11.1, 6.8, 4.3	75.2	4.24	ddd, *J =* 11.9, 9.4, 2.6	61.4
14	1.51	m	26.5	1.76	ddq, *J =* 14.5, 9.4, 7.7	28.4
	1.45	m		2.15	dddd, *J =* 14.5, 10.2, 7.7, 2.6	
15	1	t, *J =* 6.8	10.1	1.04	t, *J =* 7.7	11.2

^a^ **1** was measured in CDCl_3_; ^b^ **2** was measured in C_6_D_6_.

## Supplementary Materials

The following are available online: Figure S1a–S1r: 1D and 2D-NMR spectra for Isolaurenidificin (**1**), Figure S2a–S2p: 1D and 2D-NMR spectra for Bromlaurenidificin (**2**).
