# Supplementary material for: Isolaurenidificin and Bromlaurenidificin, Two New C15-Acetogenins from the Red Alga Laurencia obtusa
Source: Molecules. 2017 May 15;22(5):807. doi: 10.3390/molecules22050807 (PMC6154321; doi:10.3390/molecules22050807)
Supplement: Supplementary file 1 [file molecules-22-00807-s001.pdf]

SUPPLEMENTARY MATERIAL

Isolaurenidificin and Bromlaurenidificin, two new C_{15}-acetogenins from the red alga *Laurencia obtusa*

Nahed O. Bawakid[1], Walied M. Alarif[2,*], Nagla A. Albourae[3], Hajer S. Alorfi[1], Khalid O. Al-Footy[1], Sultan S. Al-Lihaibi[2] and Mohamed A. Ghandourah[2]

[1] Department of Chemistry, Faculty of Science, King Abdulaziz University, PO. Box 80203, Jeddah 21589, Saudi Arabia; nahbaw@kau.edu.sa (N.O.B.); halaorfi@kau.edu.sa (H.S.A.); Kalfooti@kau.edu.sa (Kh.O.A.).

[2] Department of Marine Chemistry, Faculty of Marine Sciences, King Abdulaziz University, PO.Box 80207, Jeddah 21589, Saudi Arabia; sallihaibi@kau.edu.sa (S.S.A.); mghandourah@kau.edu.sa (M.H.Gh.)

[3] Department of Biology, Faculty of Sciences, King Abdulaziz University, PO. Box 80207, Jeddah 21589, Saudi Arabia; nalbourae@gmail.com (N.A.A.)

* Correspondence: walied1737@yahoo.com; welaref@kau.edu.sa. Tel.: +966- 56-0352-034.

Isolaurenidificin and Bromlaurenidificin, two new C_{15}-acetogenins from the red alga *Laurencia obtusa*

Abstract

Chromatographic investigation of the CH_2Cl_2/MeOH extract of the Red Sea red alga *Laurencia obtusa* gave two new hexahydrofuro[3,2-b]furan-based C_{15}-acetogenin, namely, isolaurenidificin (**1**) and bromlaurenidificin (**2**). Their chemical structures were elucidated based on extensive analyses of their spectral data. The apoptosis-inducing or inhibiting effect of both compounds on apoptosis of peripheral blood neutrophils was studied.

Keywords**:** Marine algae; Fatty acids; halogenations; spectroscopy; Anti-inflammatory.

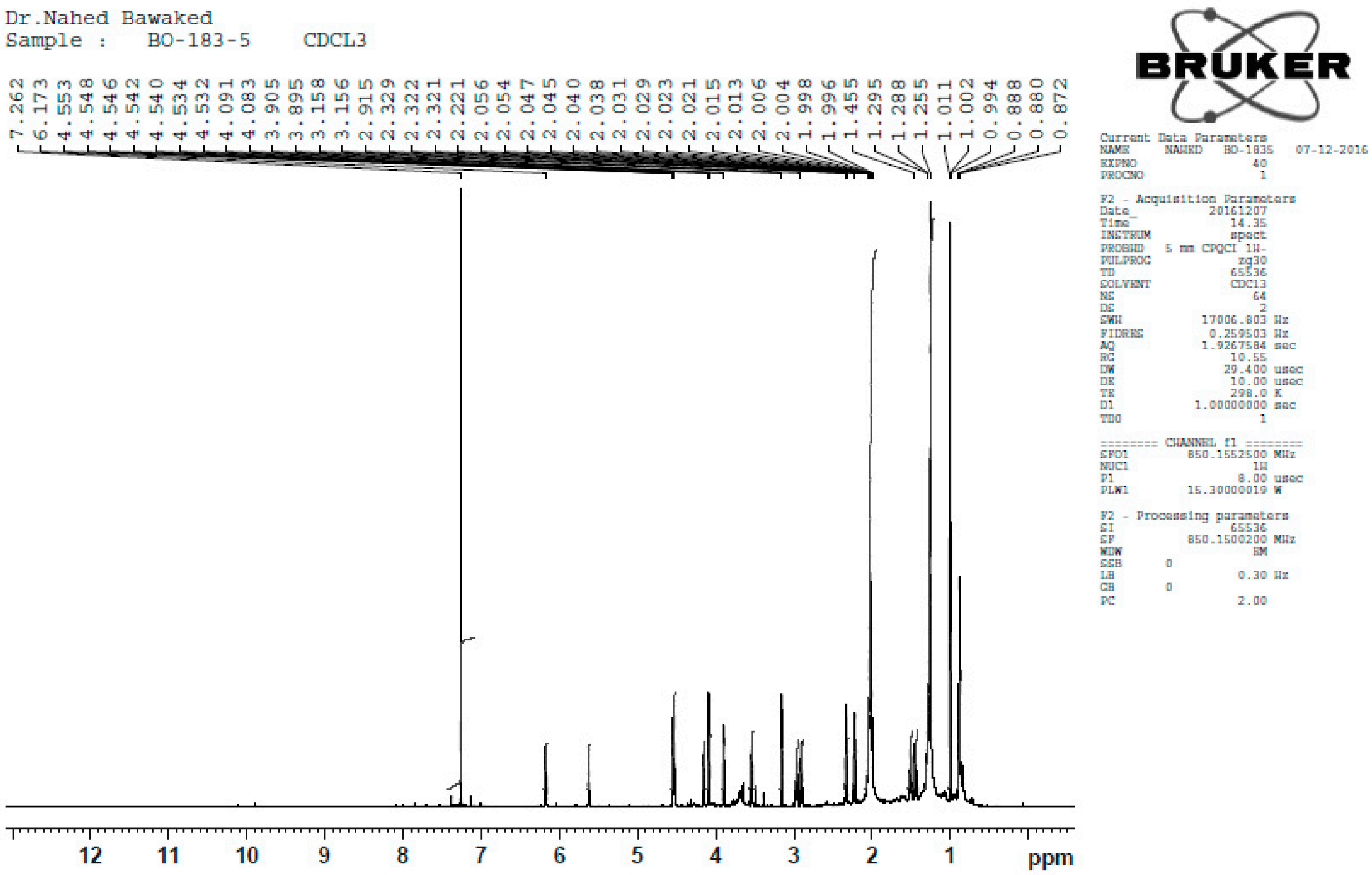

Figure S1a: ^{1}HNMR of compound 1

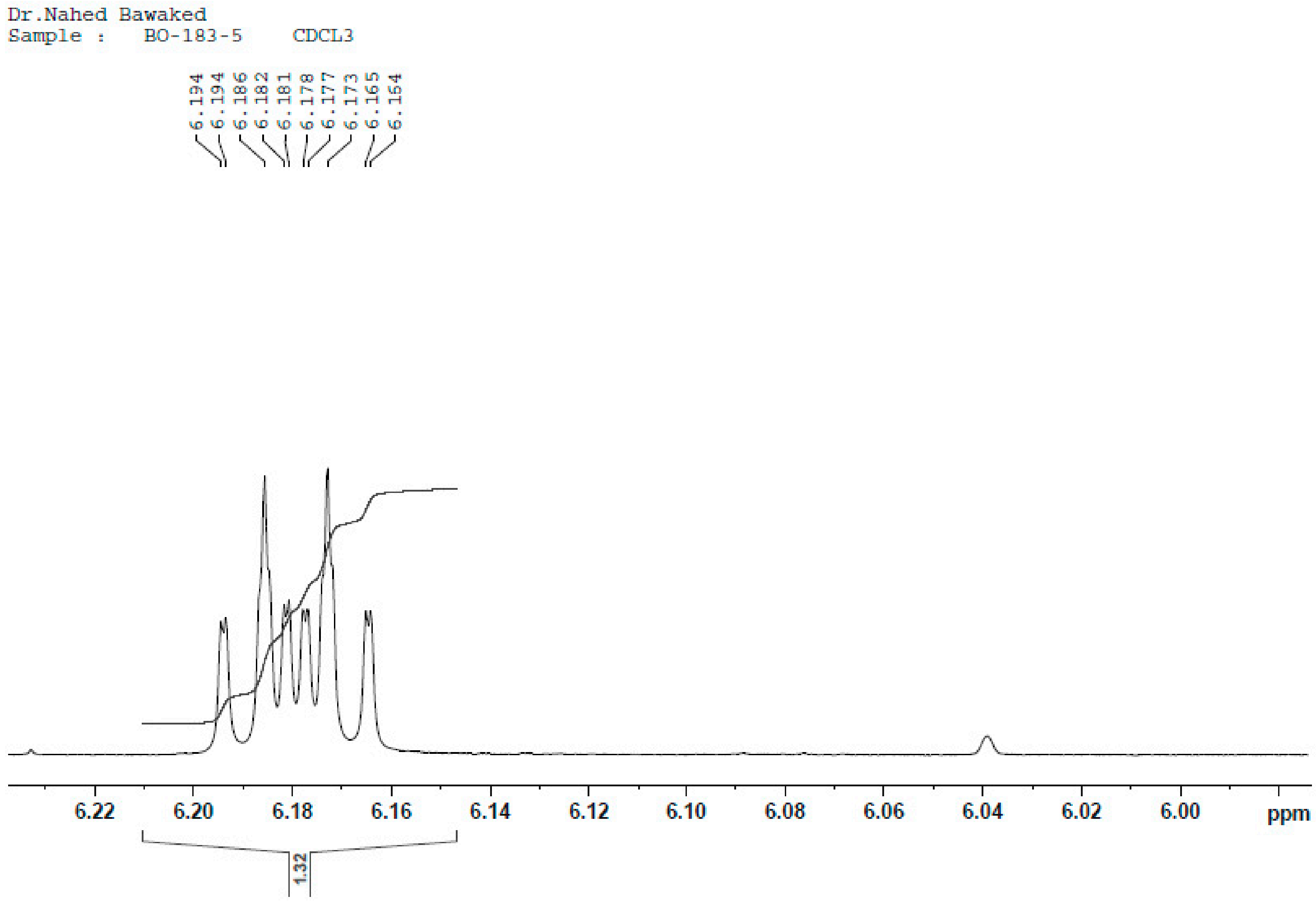

Figure S1b: ^{1}HNMR of compound 1

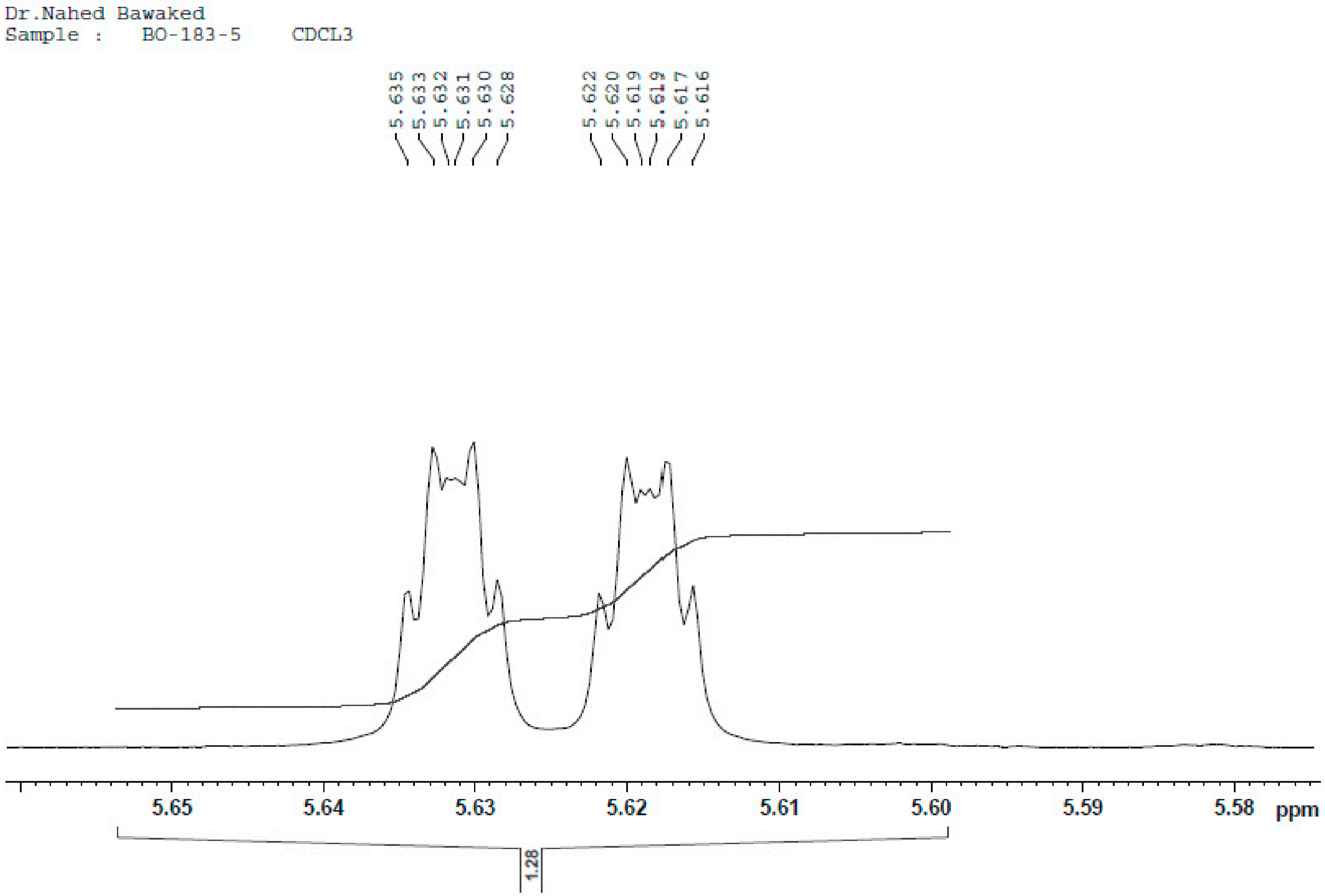

Figure S1c: ^{1}HNMR of compound 1

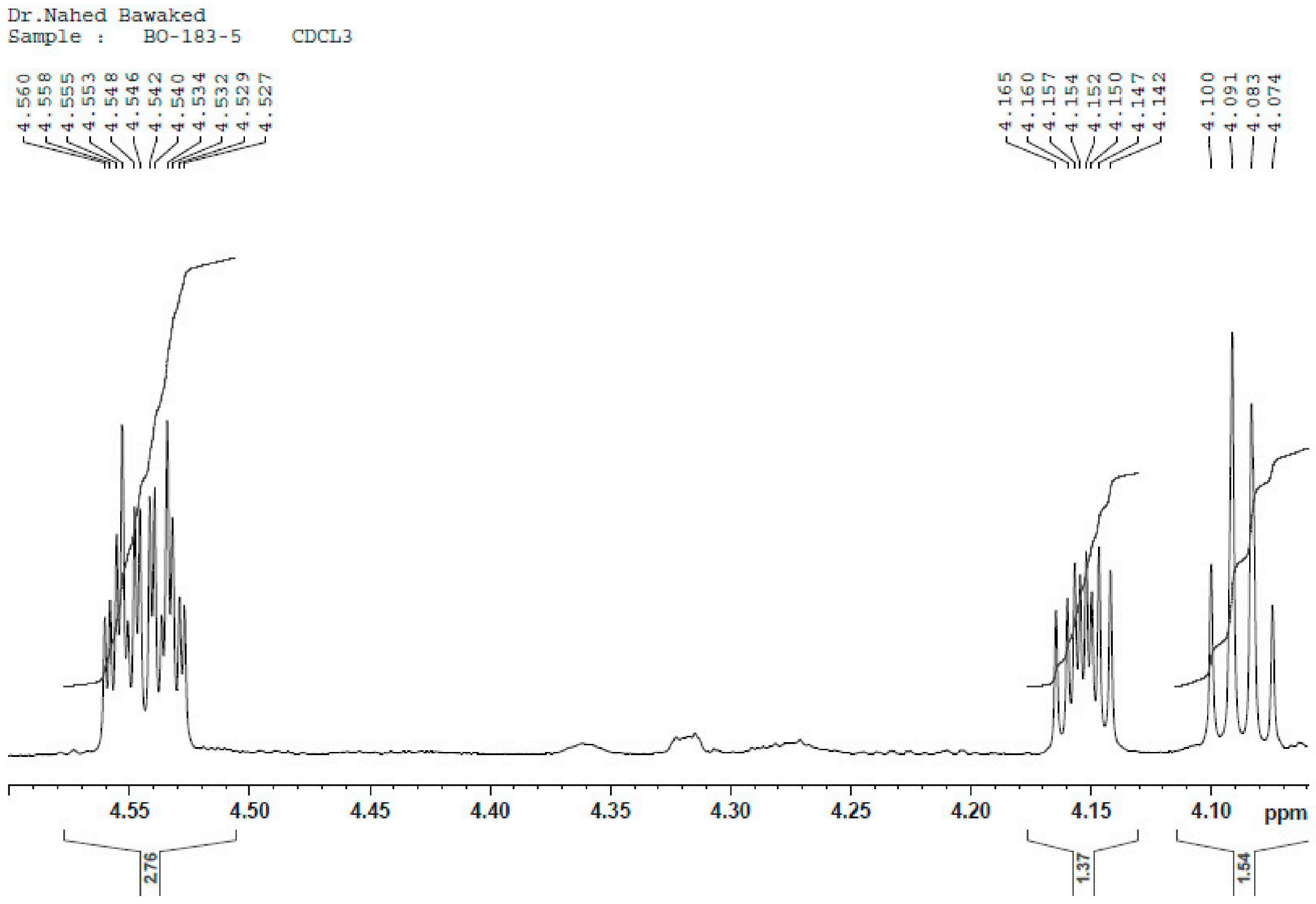

Figure S1d: ^{1}HNMR of compound 1

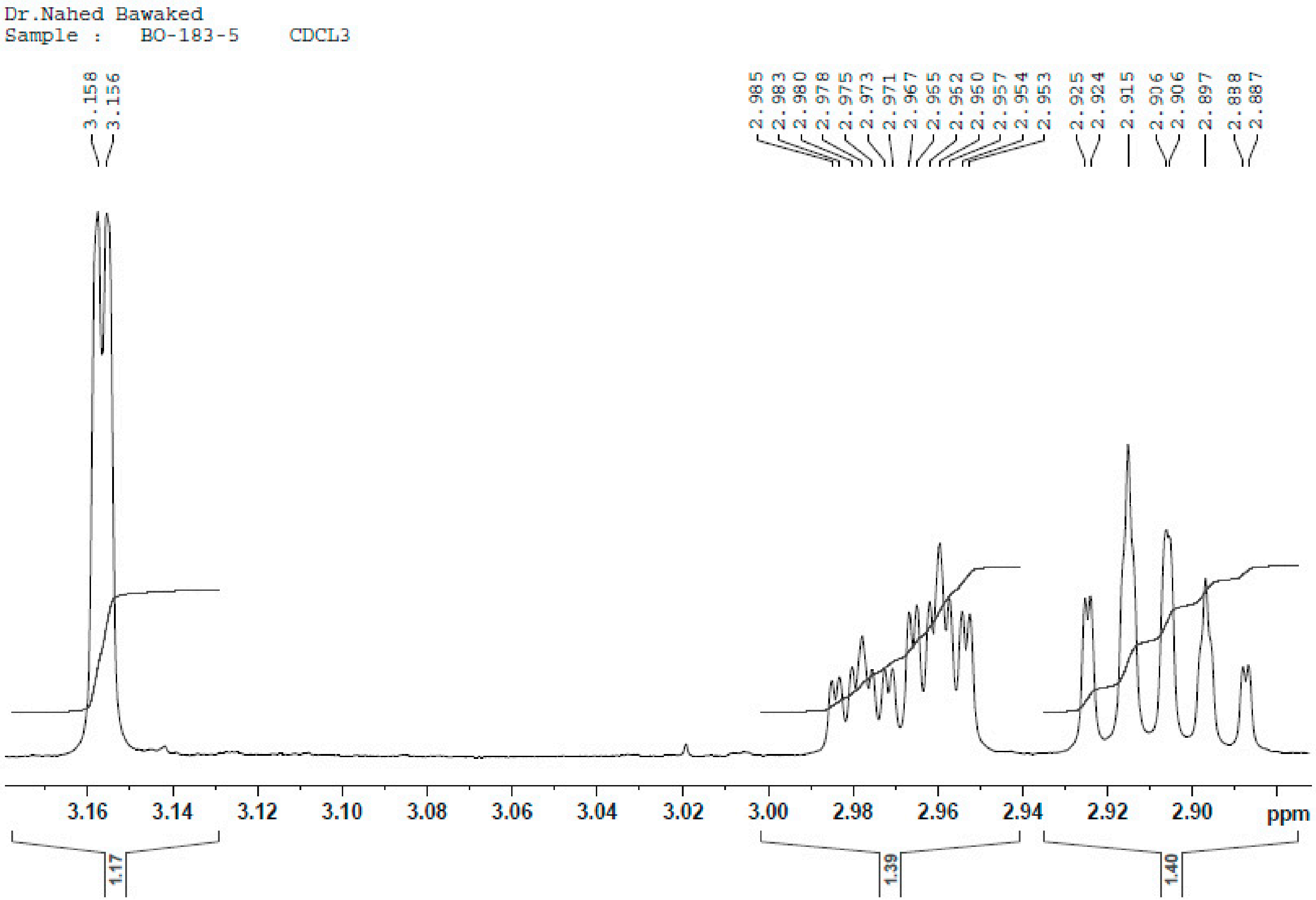

Figure S1e: ^{1}HNMR of compound 1

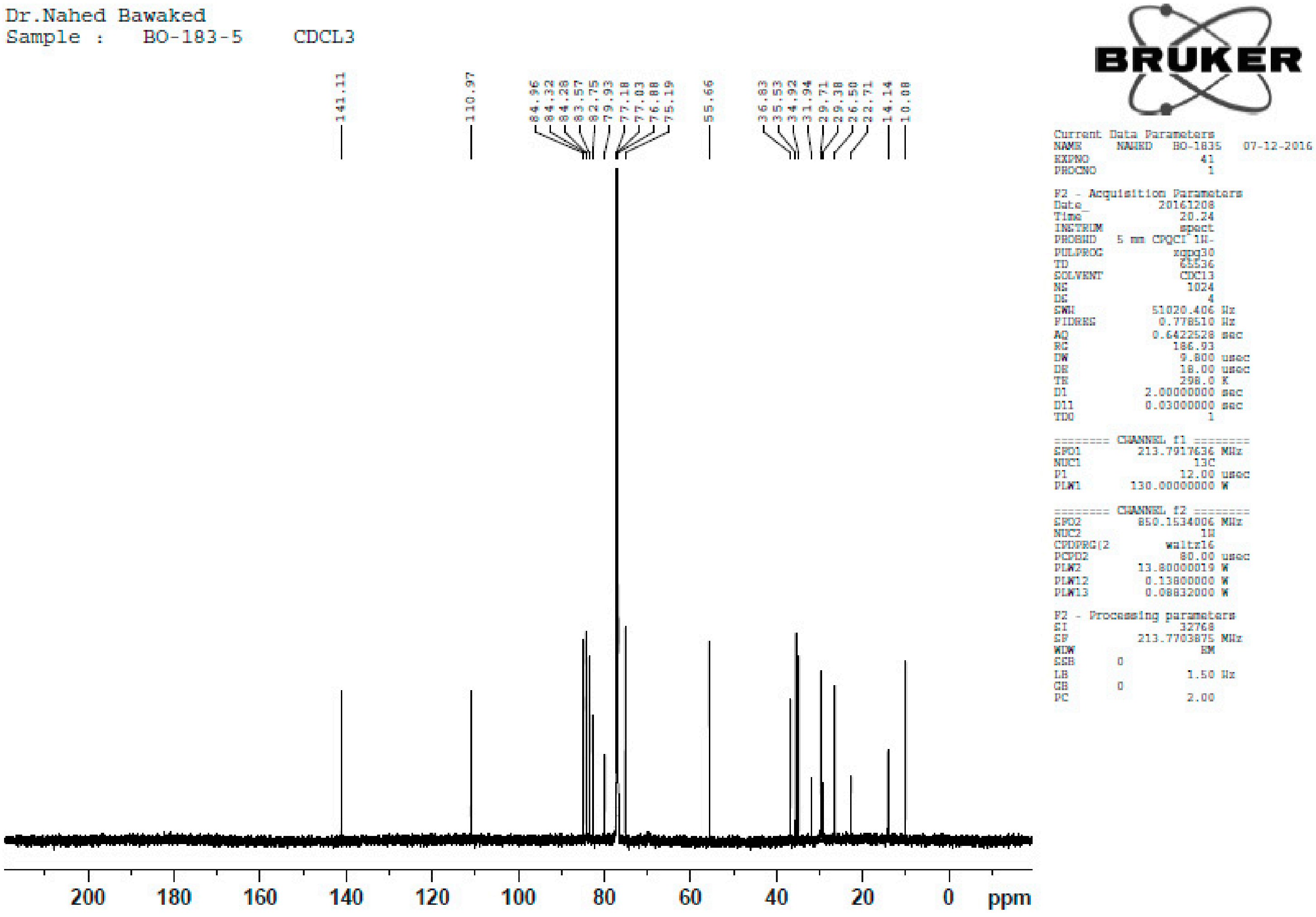

Figure S1f: ^{13}CNMR of compound 1

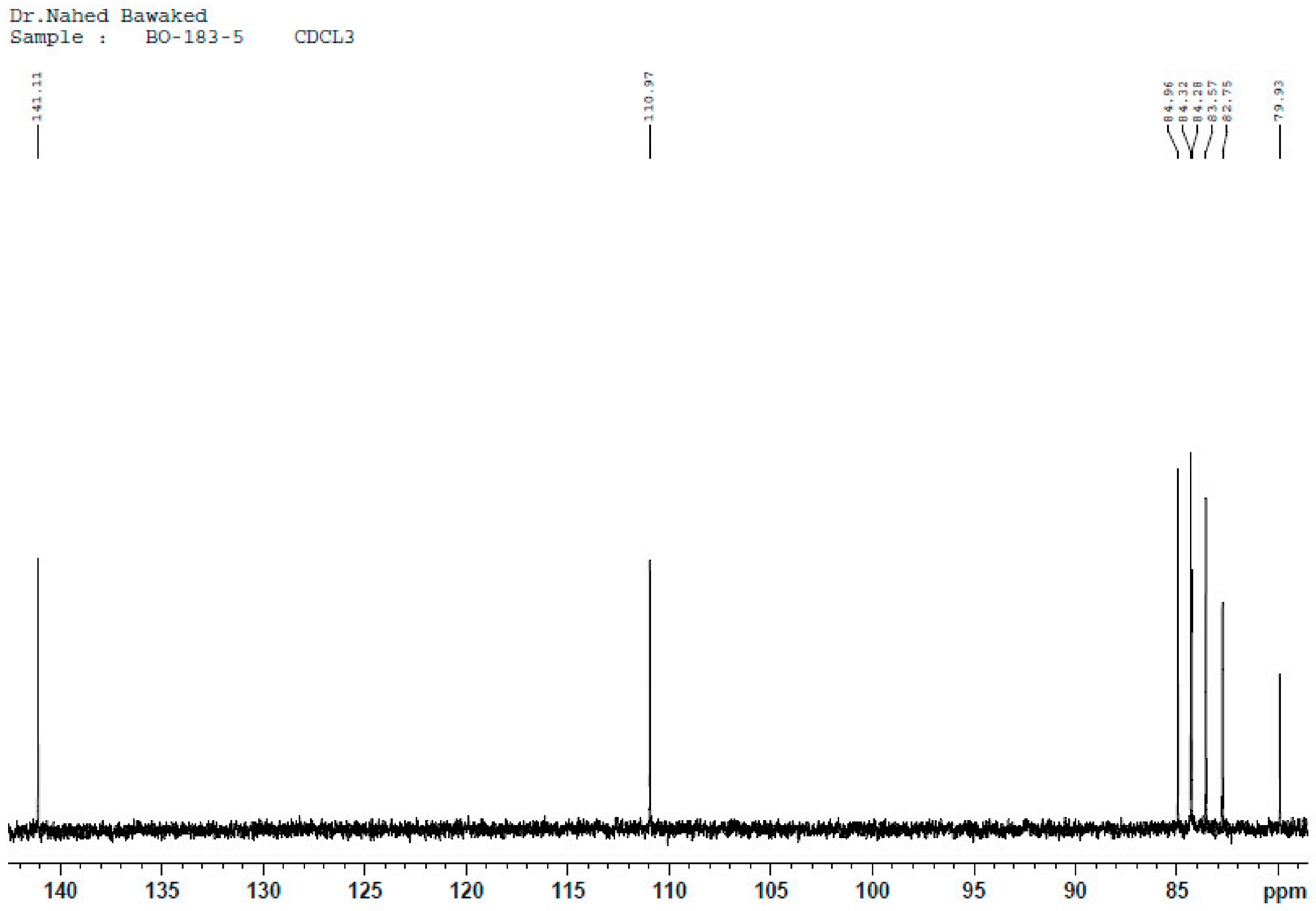

Figure S1g: ^{13}CNMR of compound 1

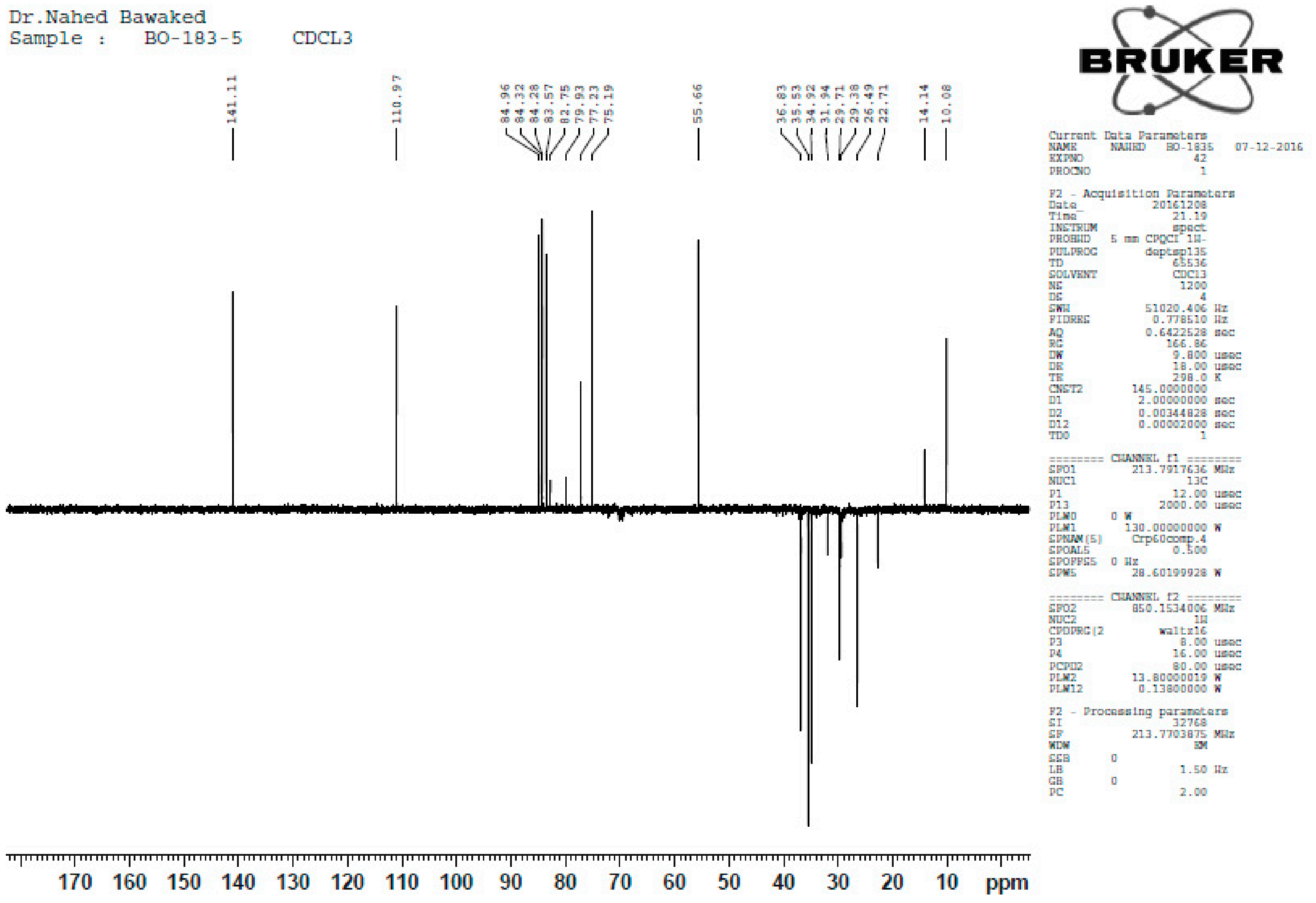

Figure S1h: DEPT NMR of compound 1

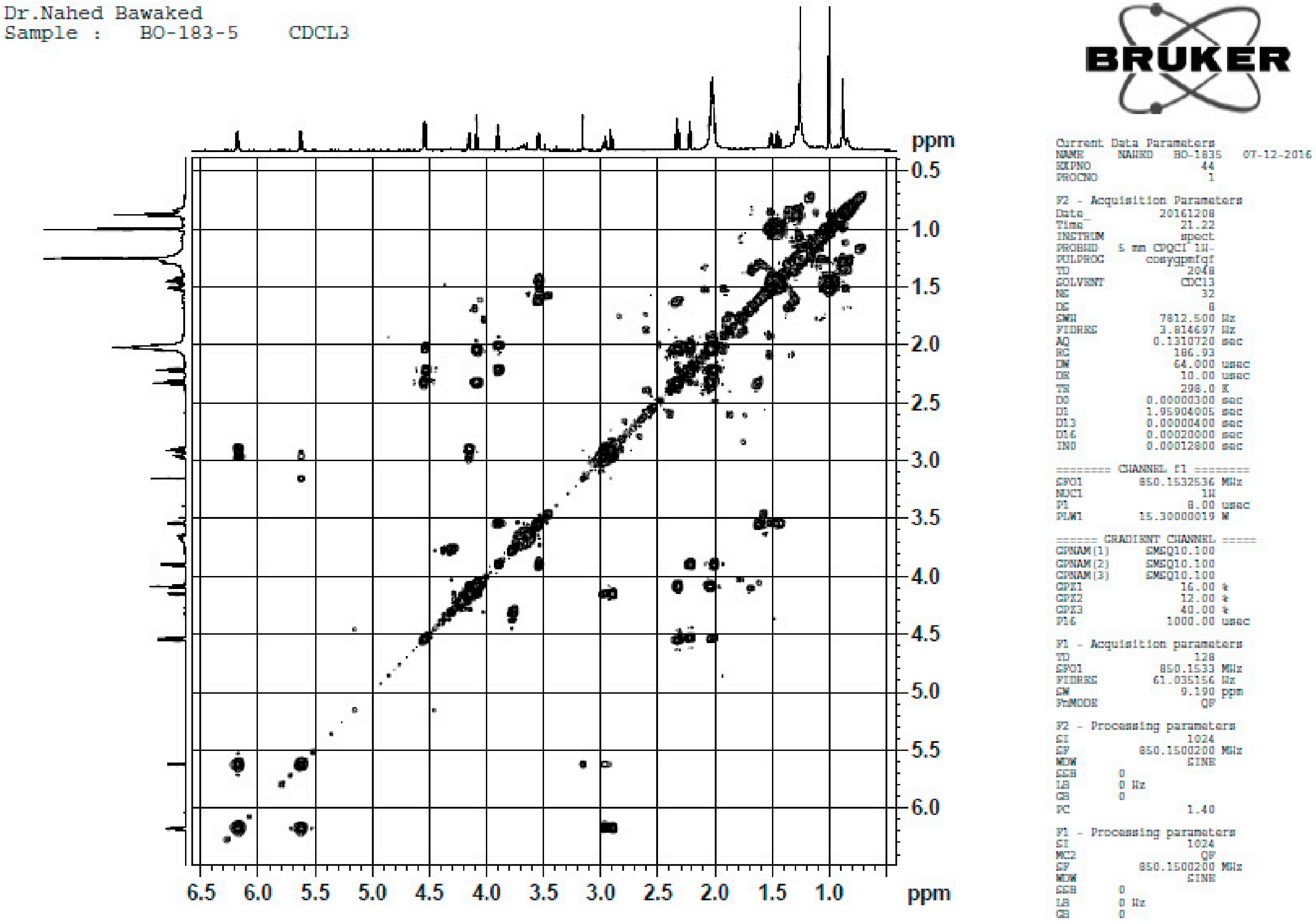

Figure S1i: COSY NMR of compound 1

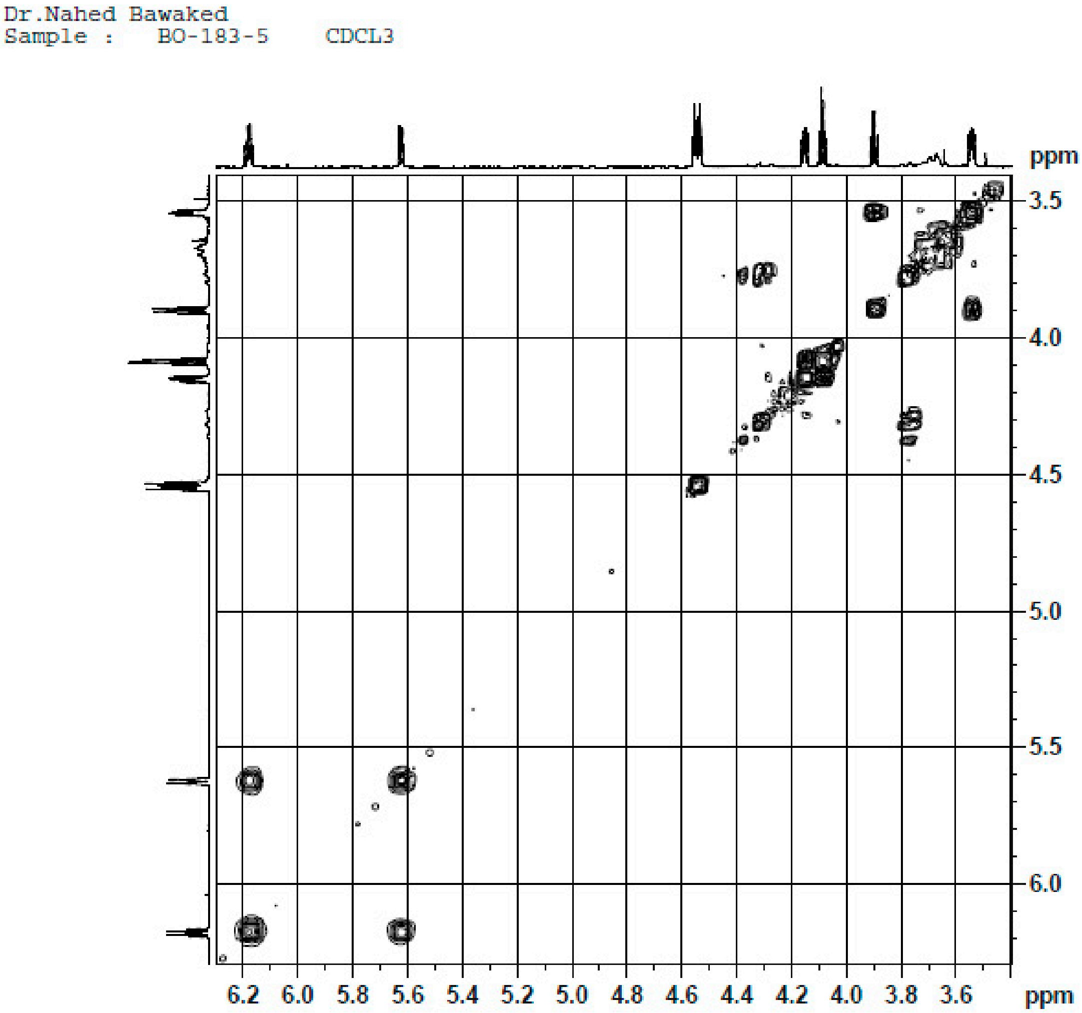

Figure S1j: COSY NMR of compound 1

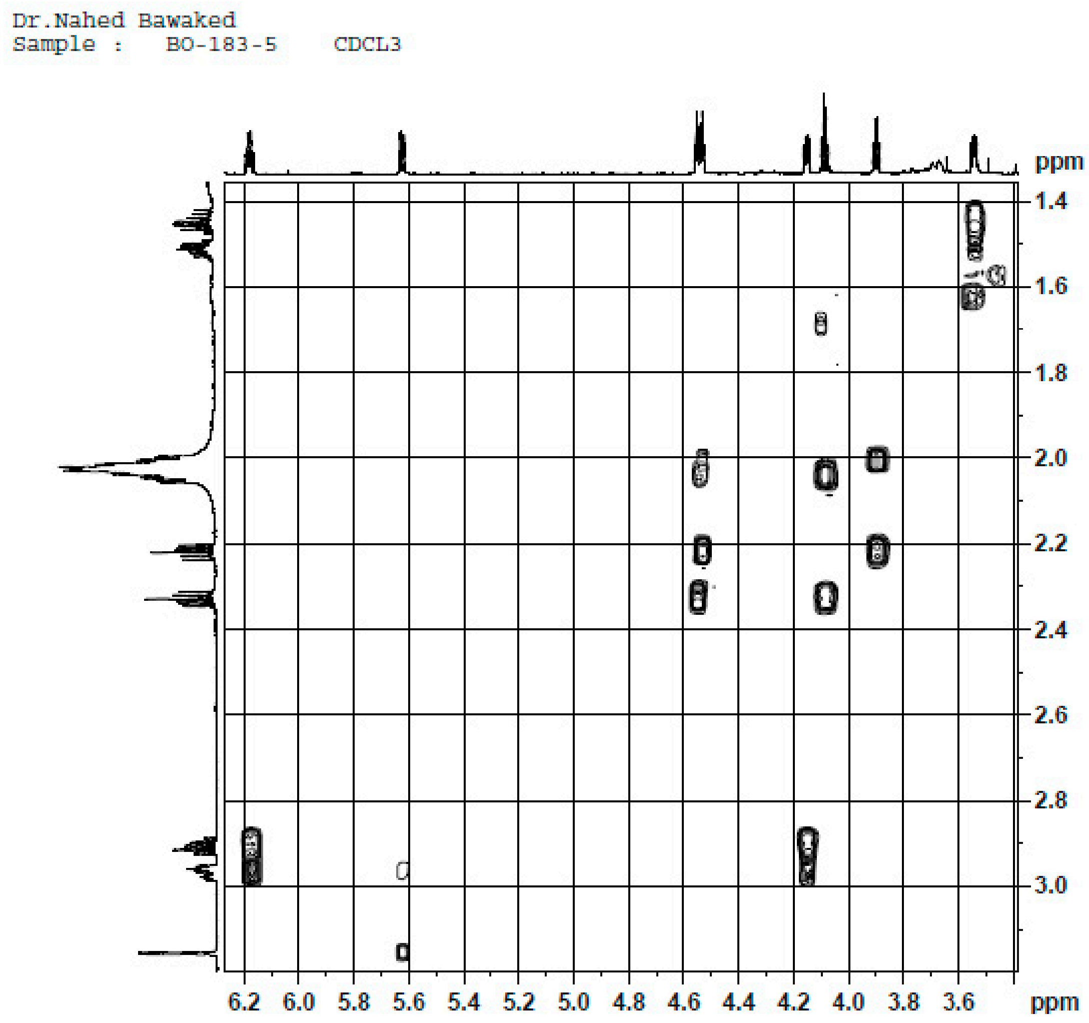

Figure S1k: COSY NMR of compound 1

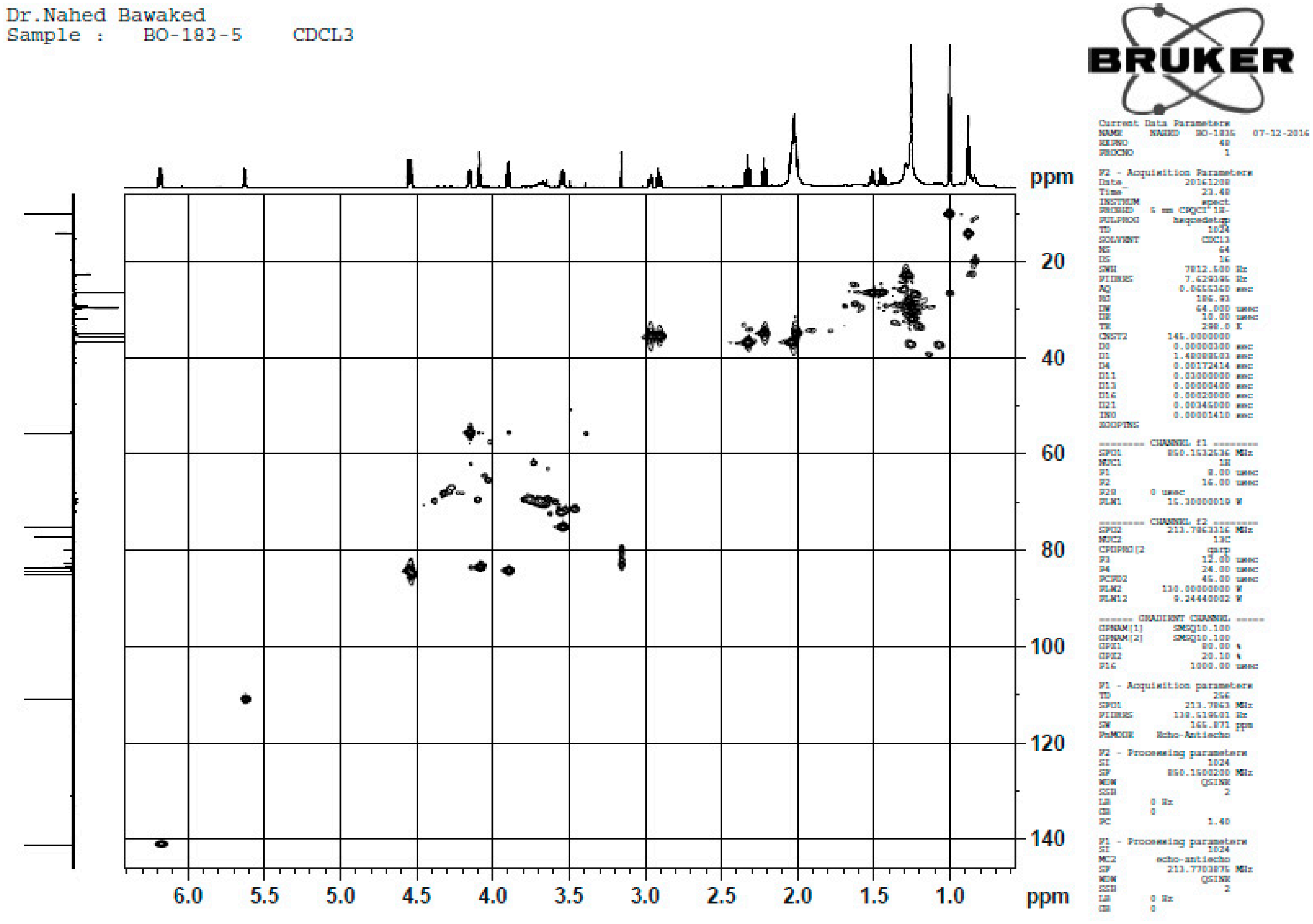

Figure S1l: HSQC NMR of compound 1

Dr.Nahed Bawaked
Sample :   BO-183-5   CDCL3

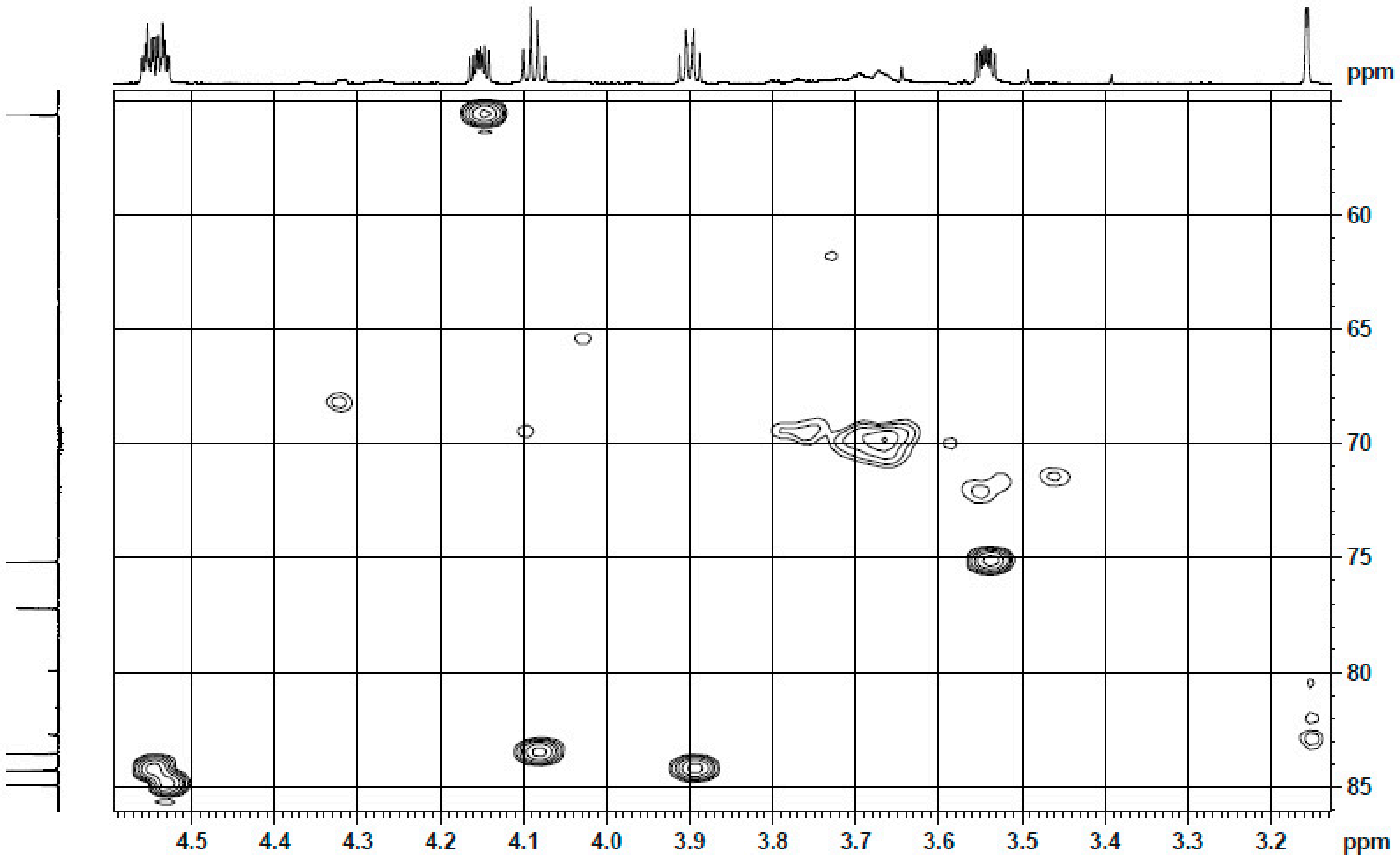

Figure S1m: HSQC NMR of compound 1

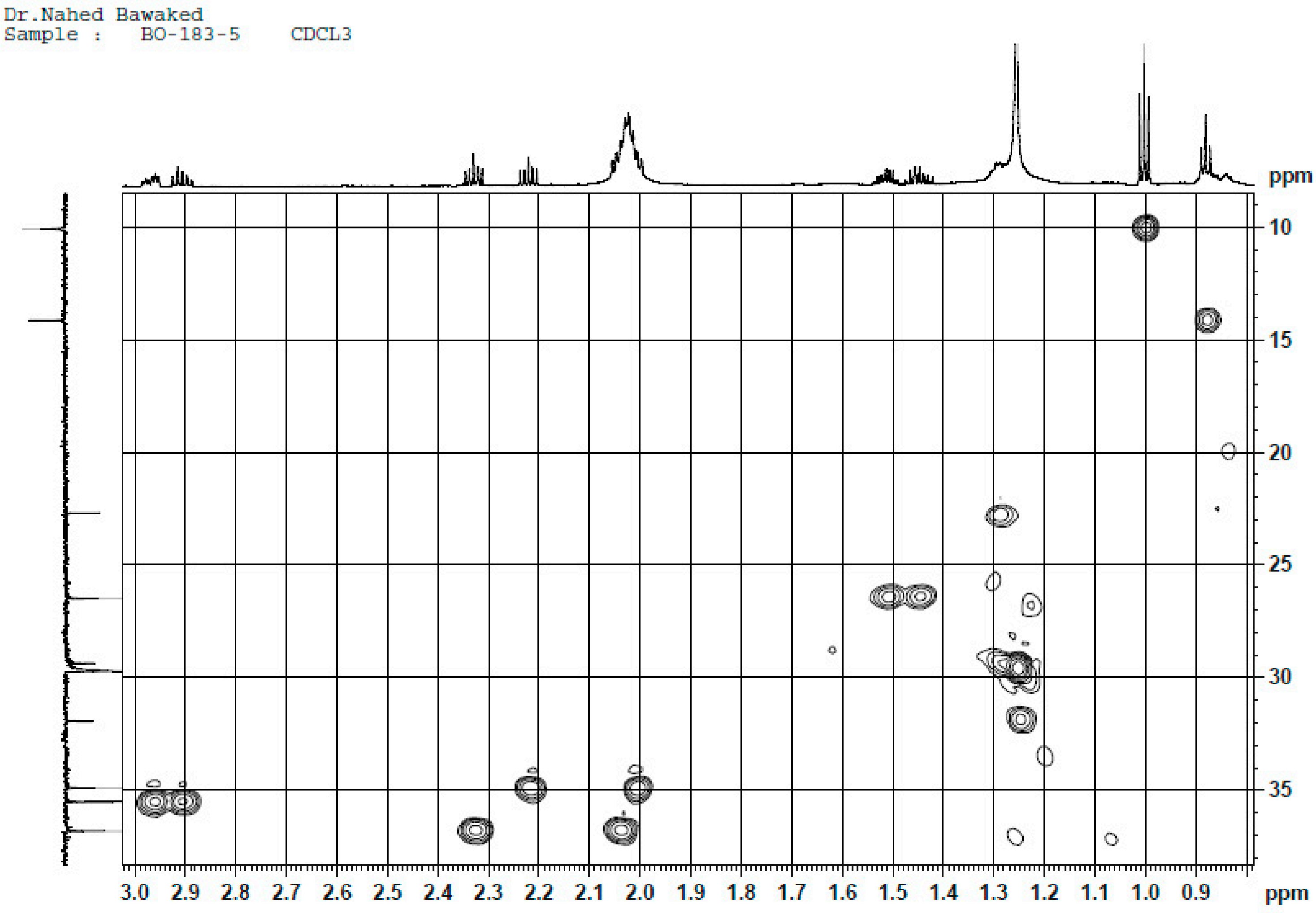

Figure S1n: HSQC NMR of compound 1

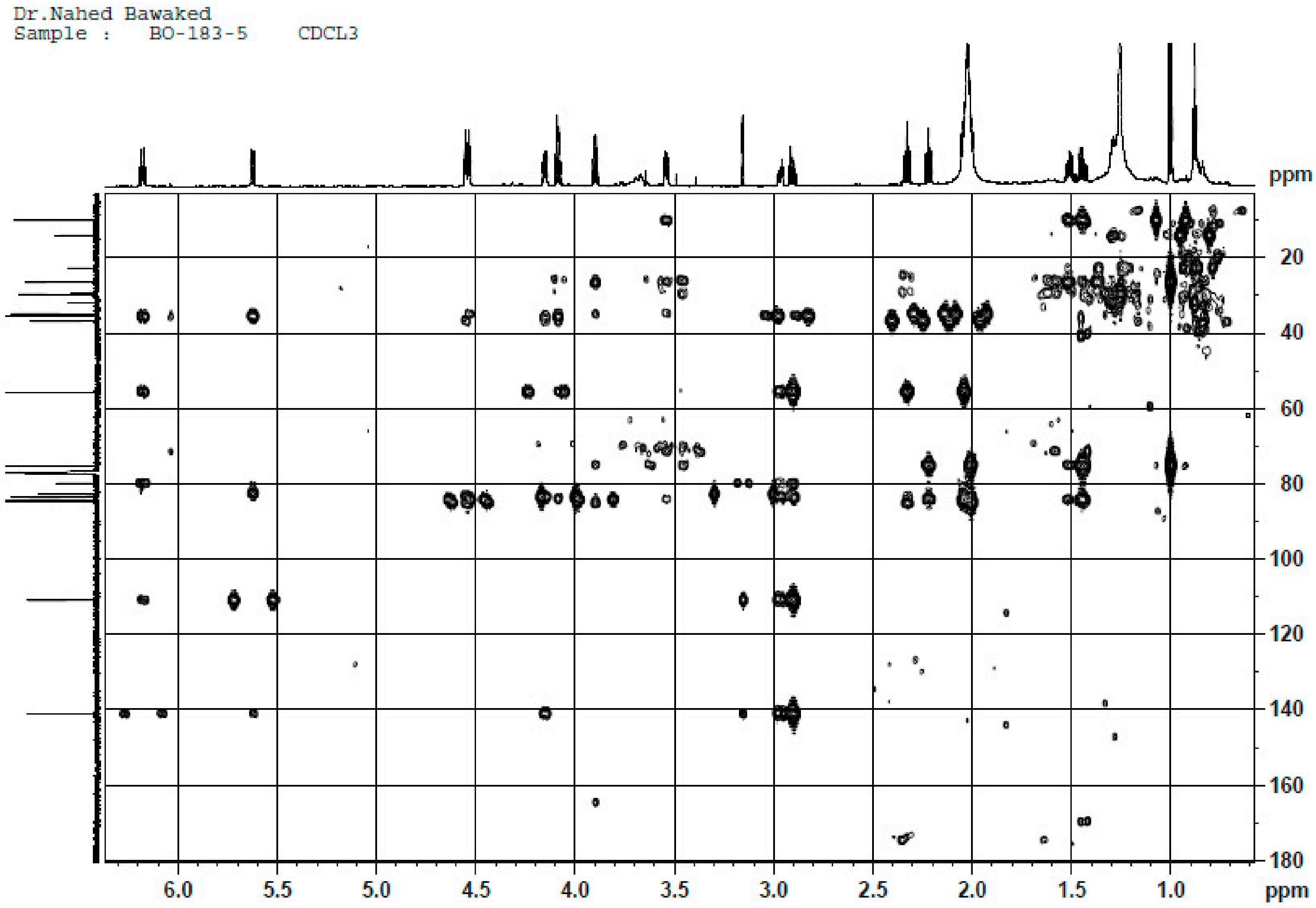

Figure S1o: HMBC NMR of compound 1

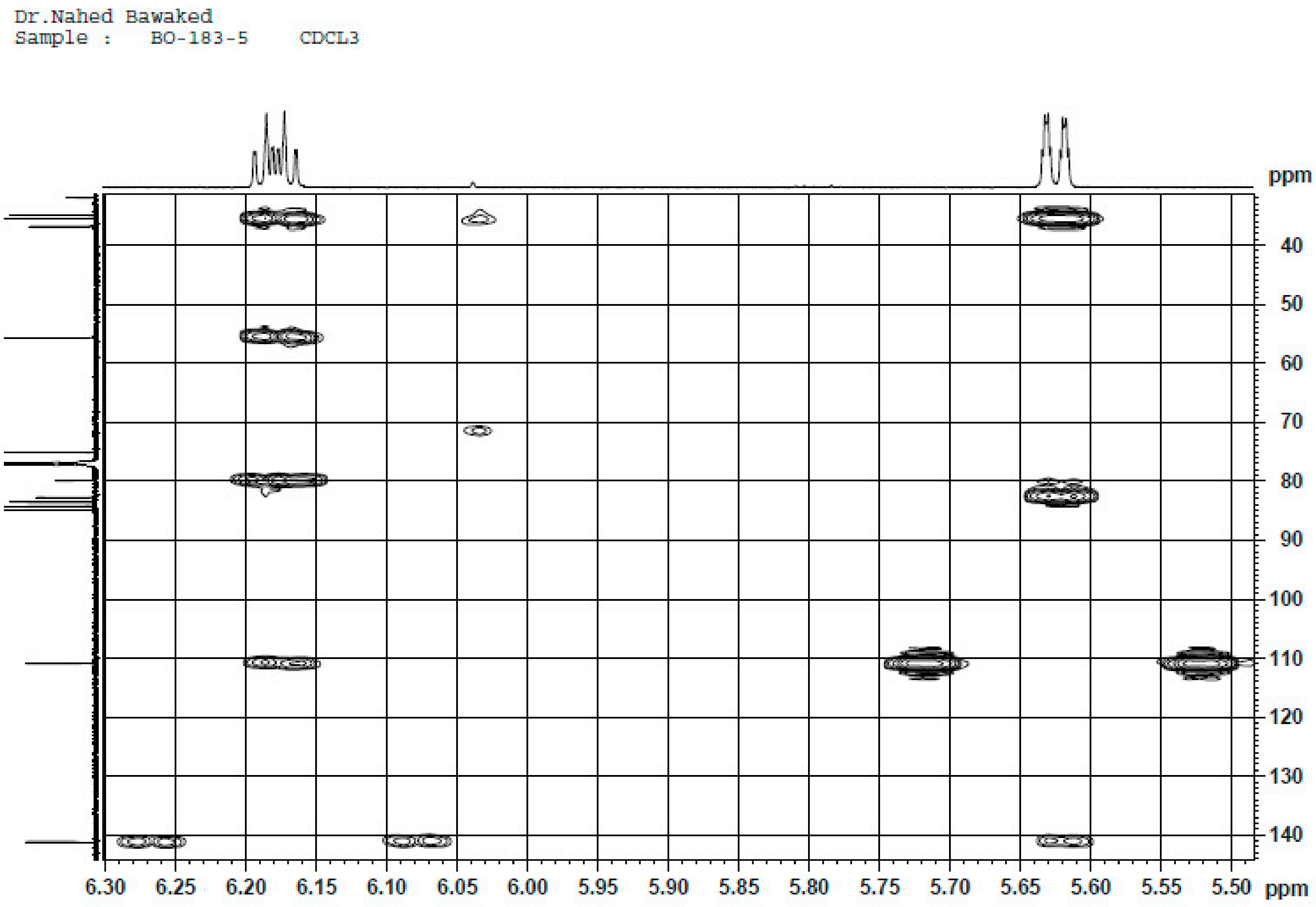

Figure S1p: HMBC NMR of compound 1

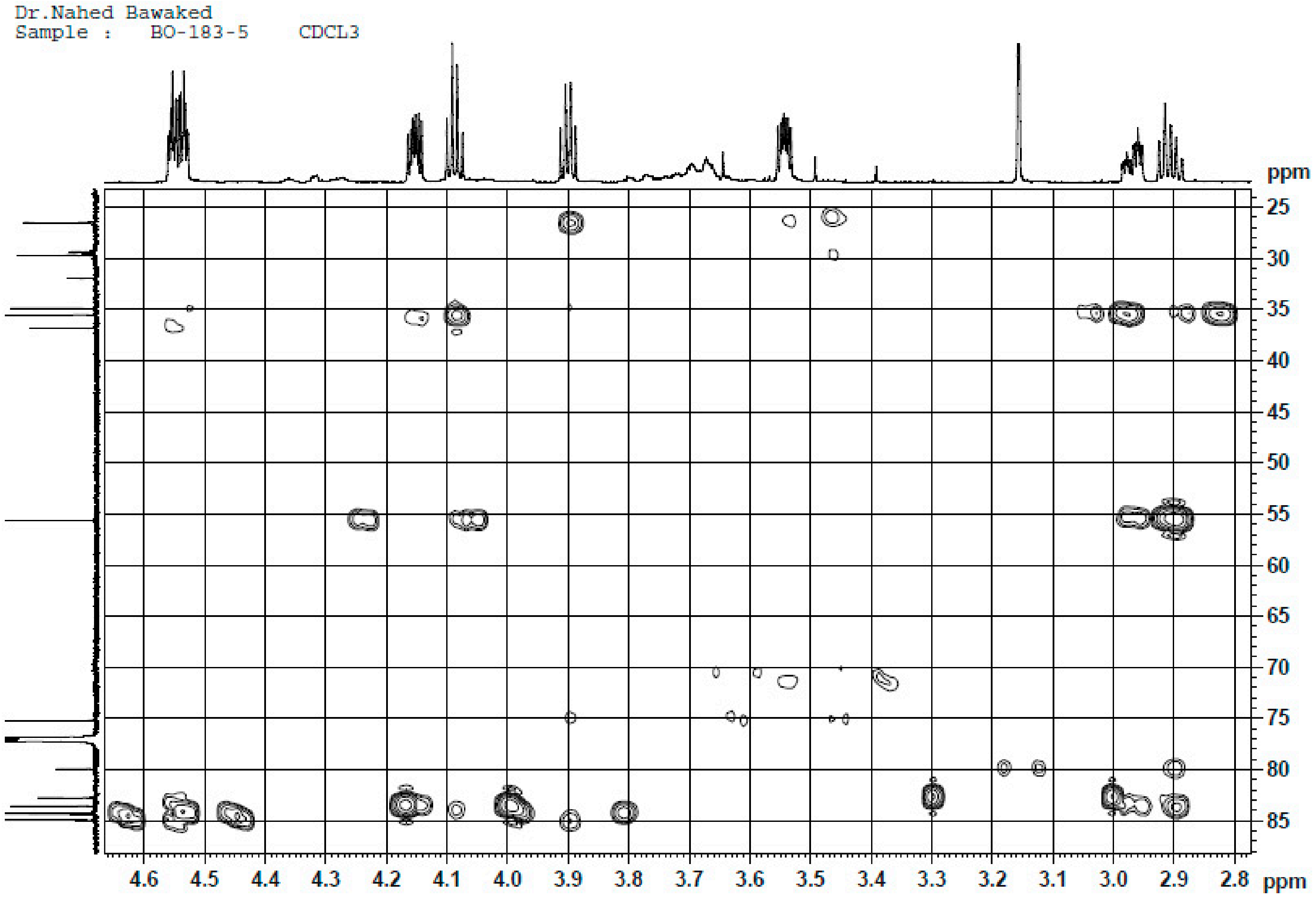

Figure S1q: HMBC NMR of compound 1

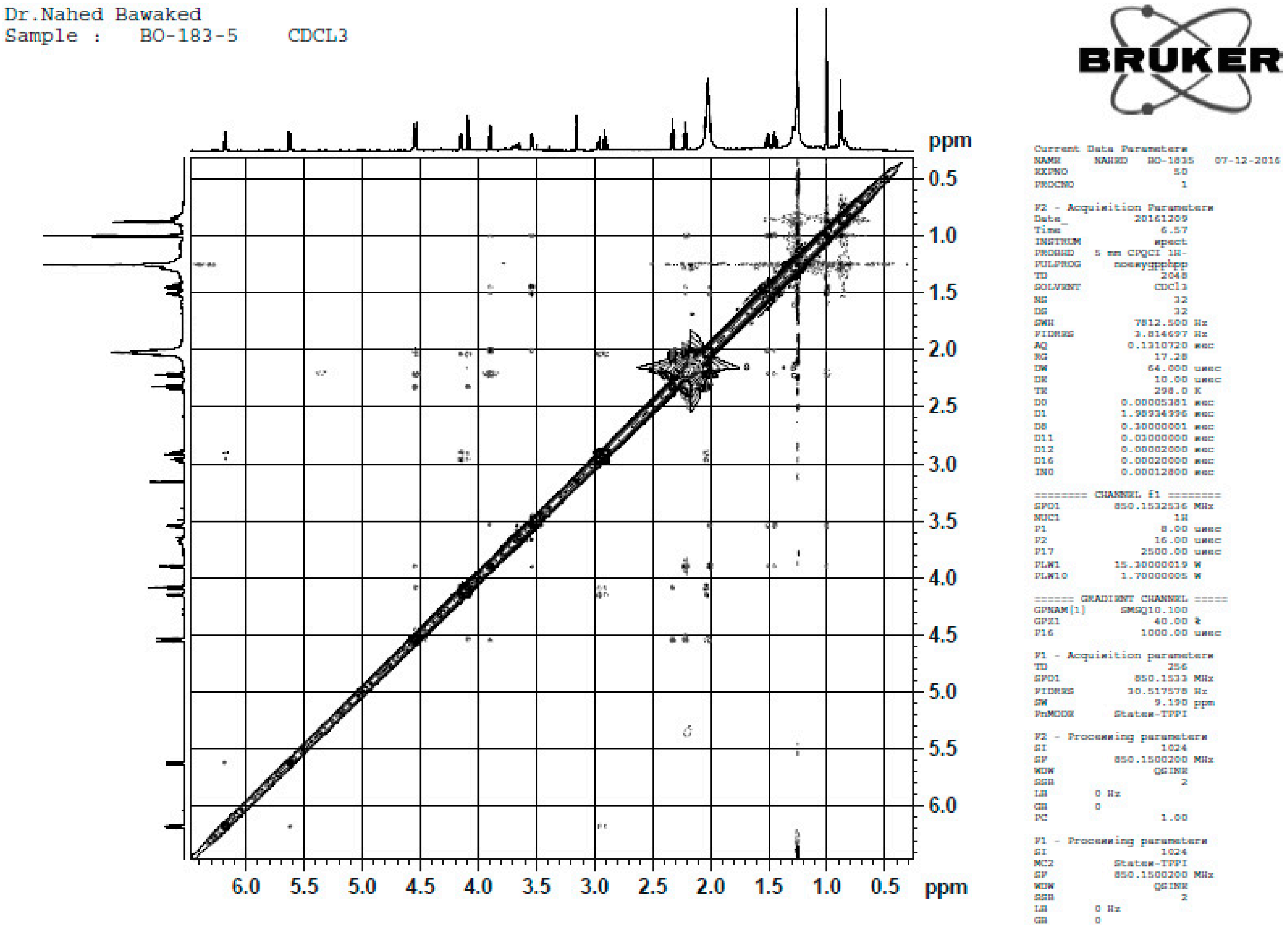

Figure S1r: NOESY NMR of compound 1

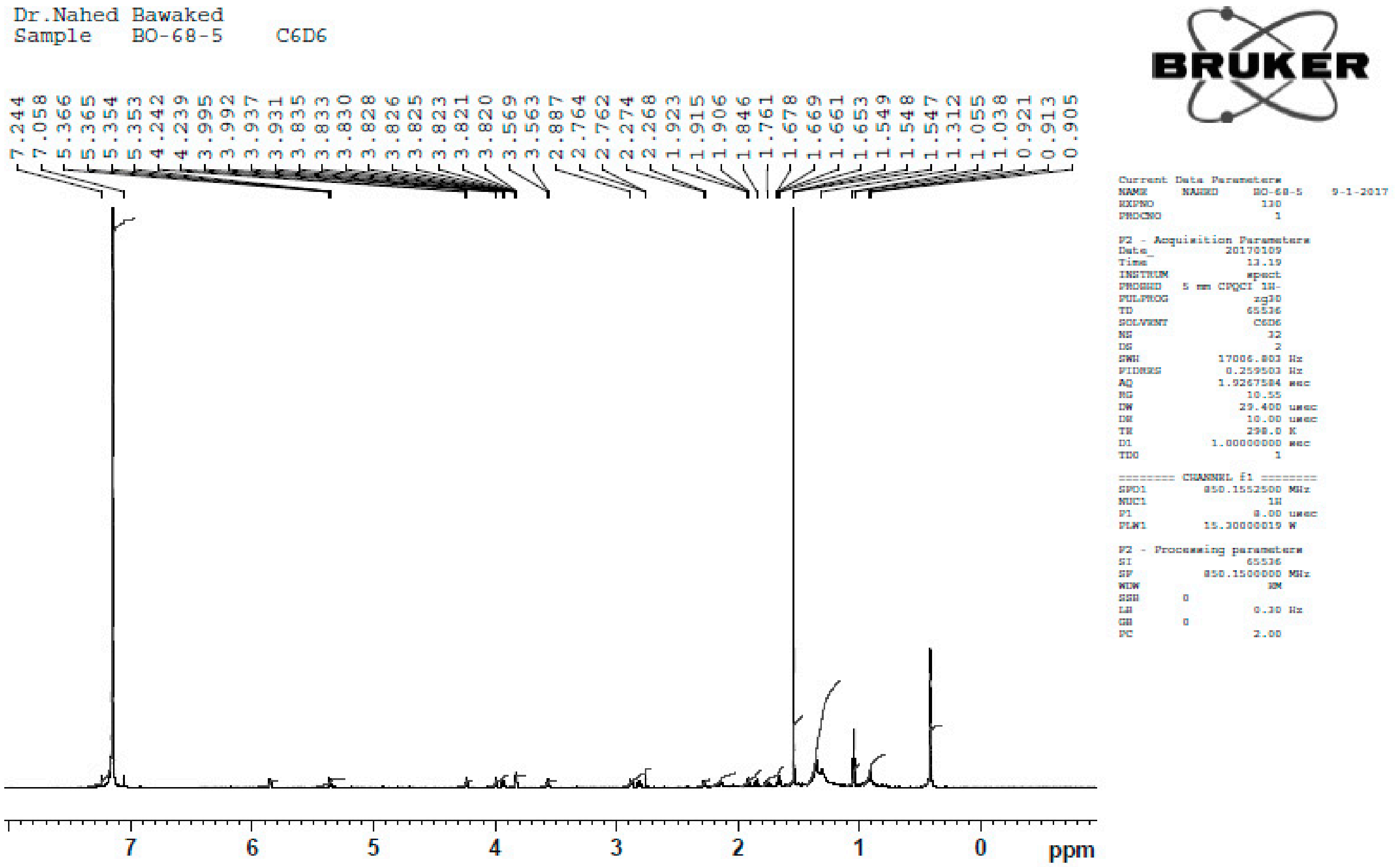

Figure S2a: ^{1}HNMR of compound 2

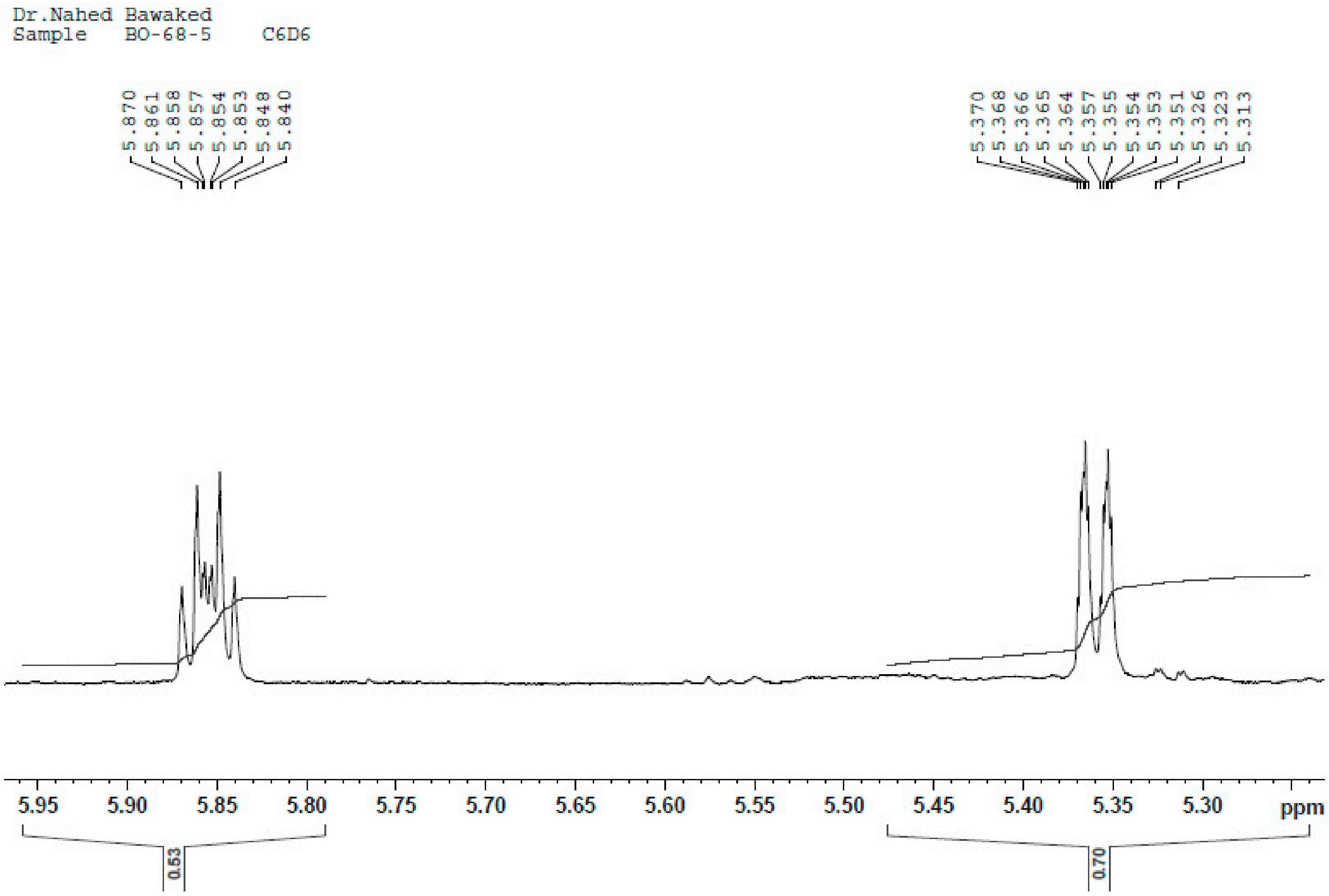

Figure S2b: ^{1}HNMR of compound 2

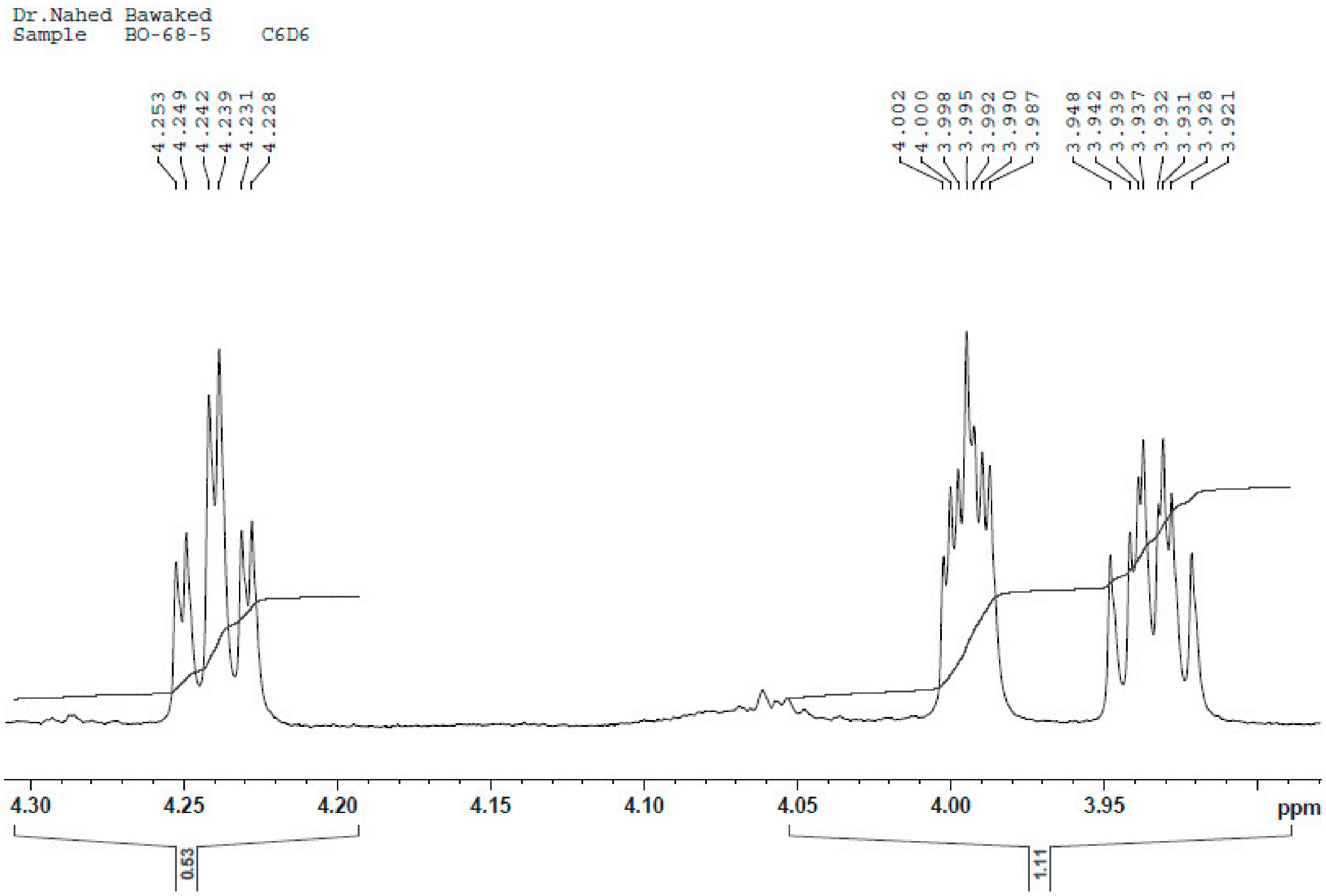

Figure S2c: ^{1}HNMR of compound 2

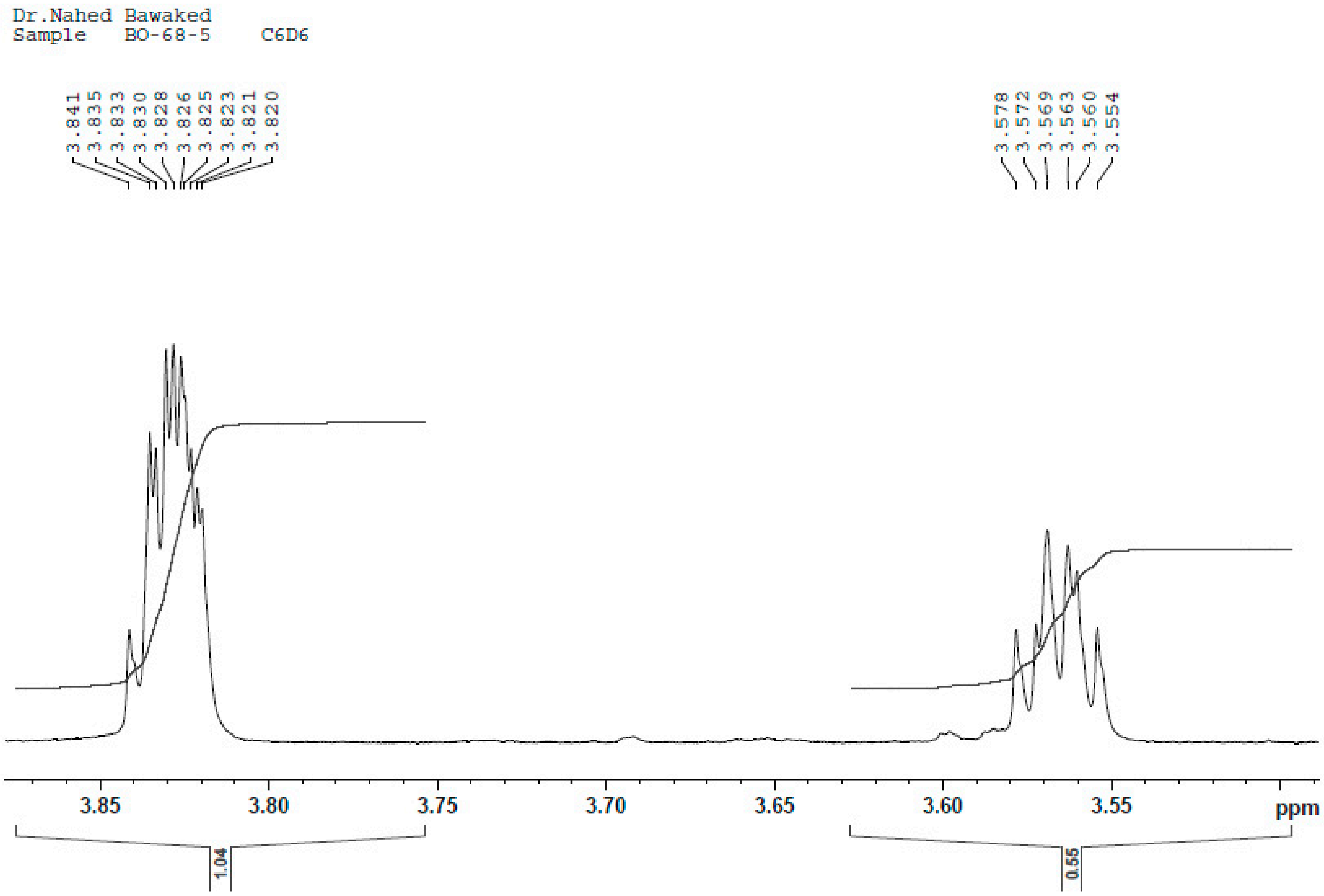

Figure S2d: ^{1}HNMR of compound 2

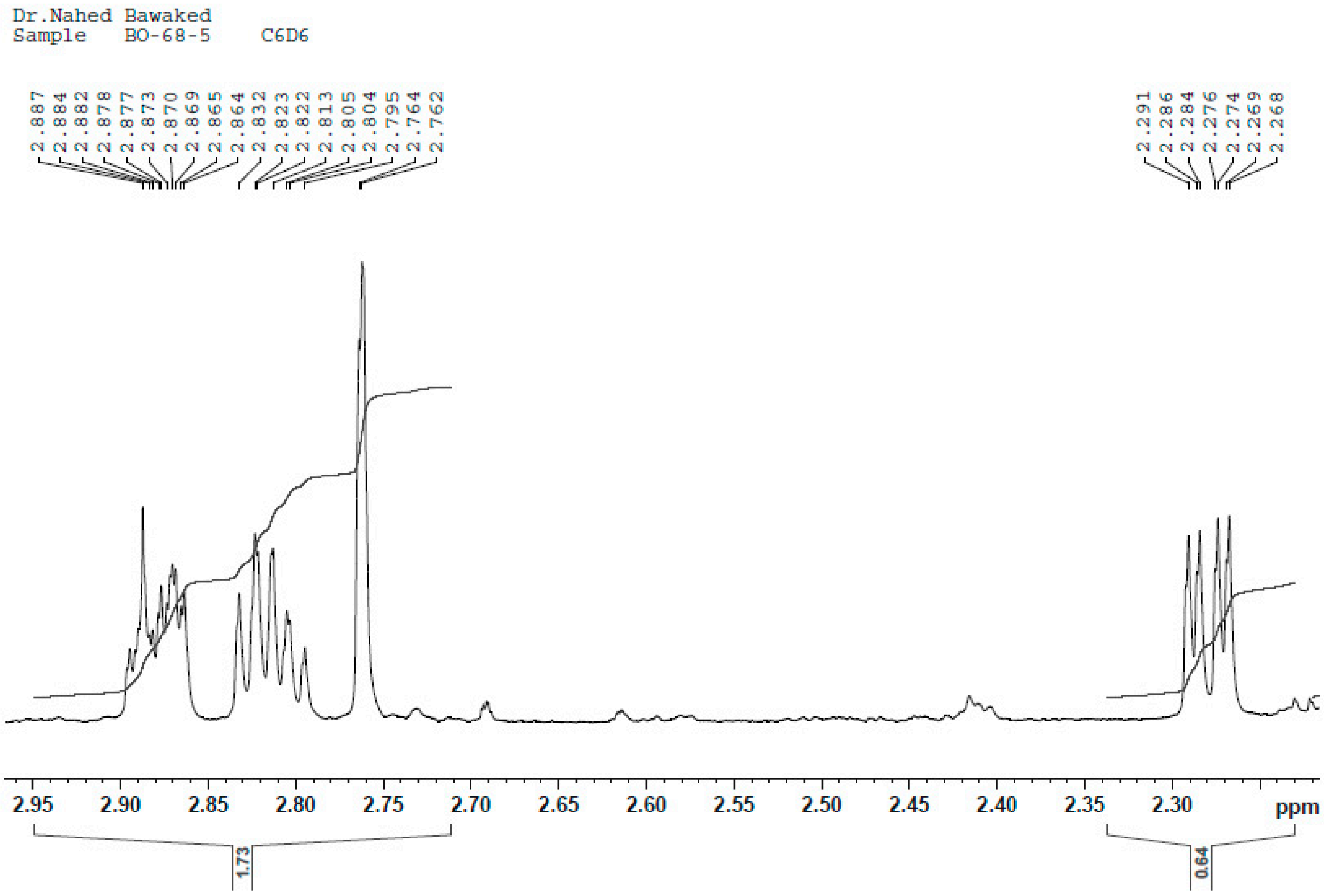

Figure S2e: ^{1}HNMR of compound 2

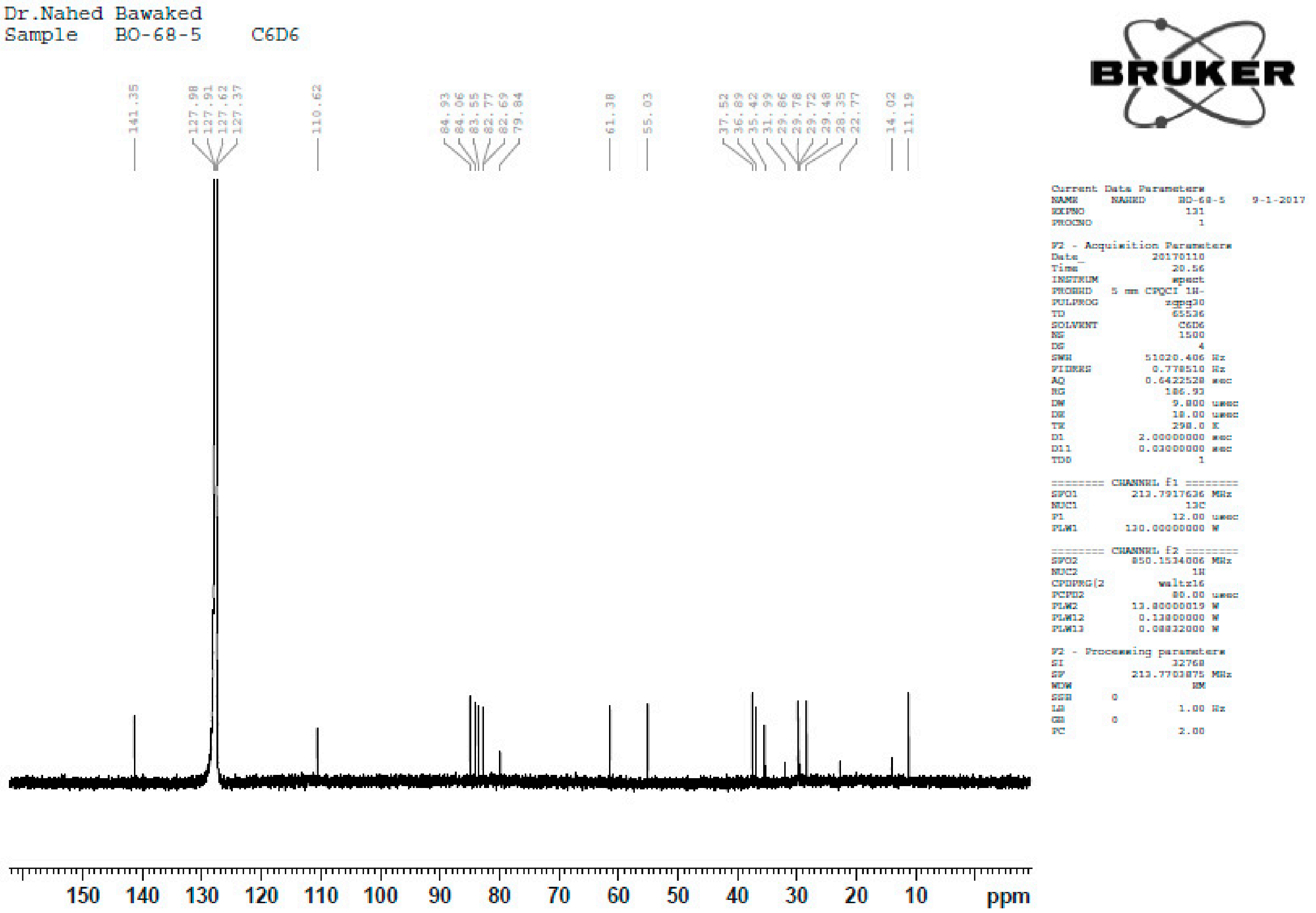

Figure S2f: ^{13}CNMR of compound 2

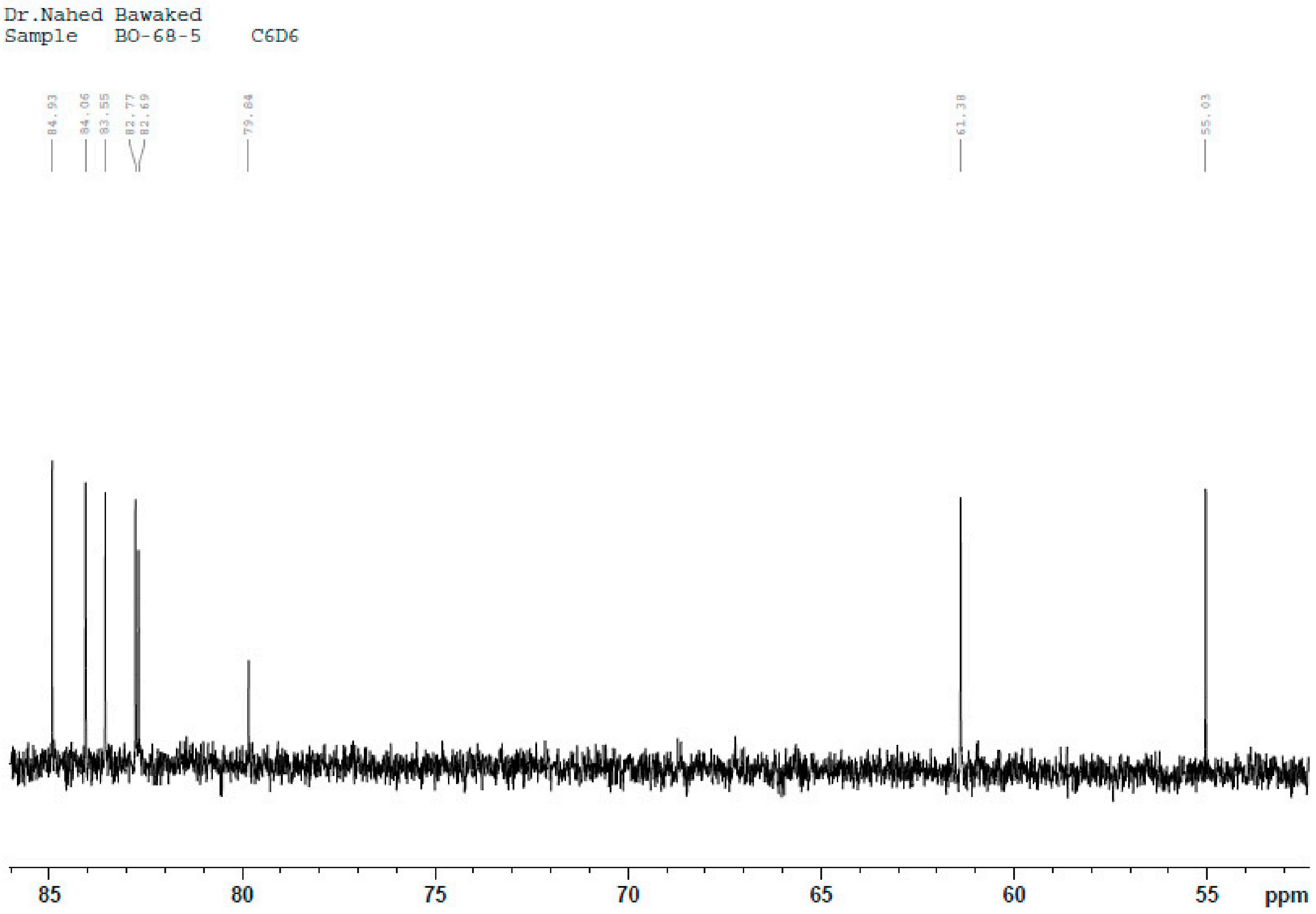

Figure S2g: ^{13}CNMR of compound 2

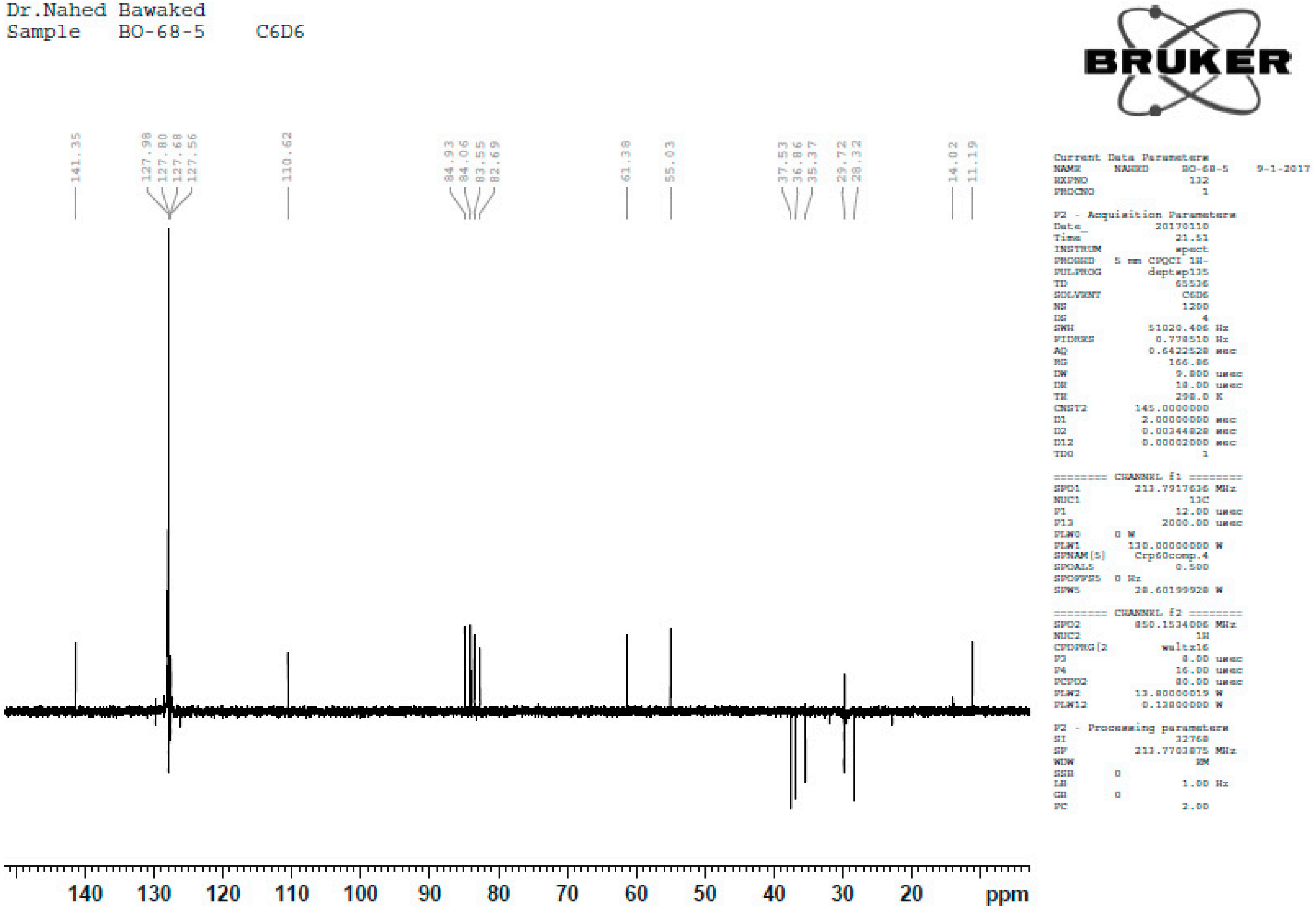

Figure S2h: DEPT NMR of compound 2

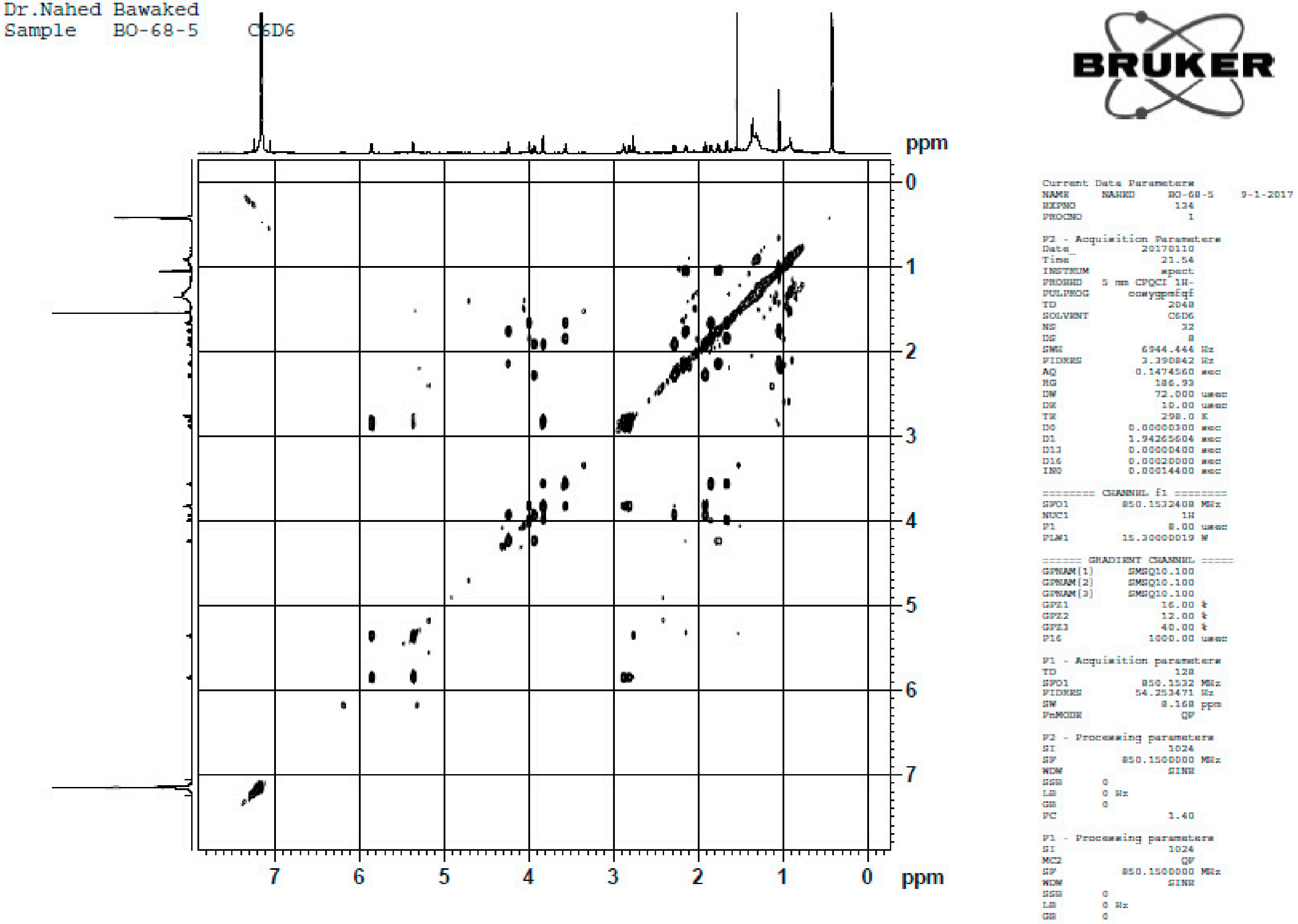

Figure S2i: COSY NMR of compound 2

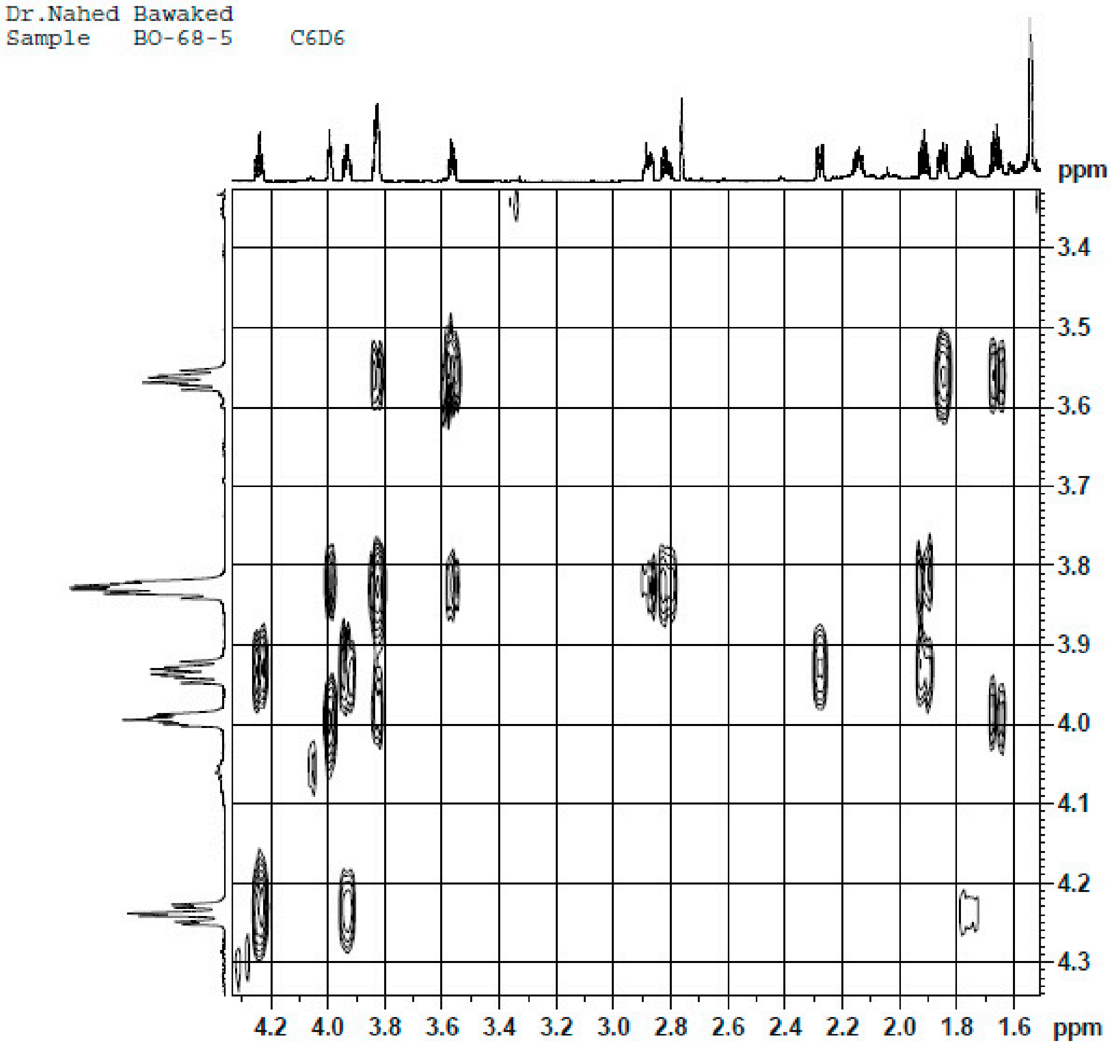

Figure S2j: COSY NMR of compound 2

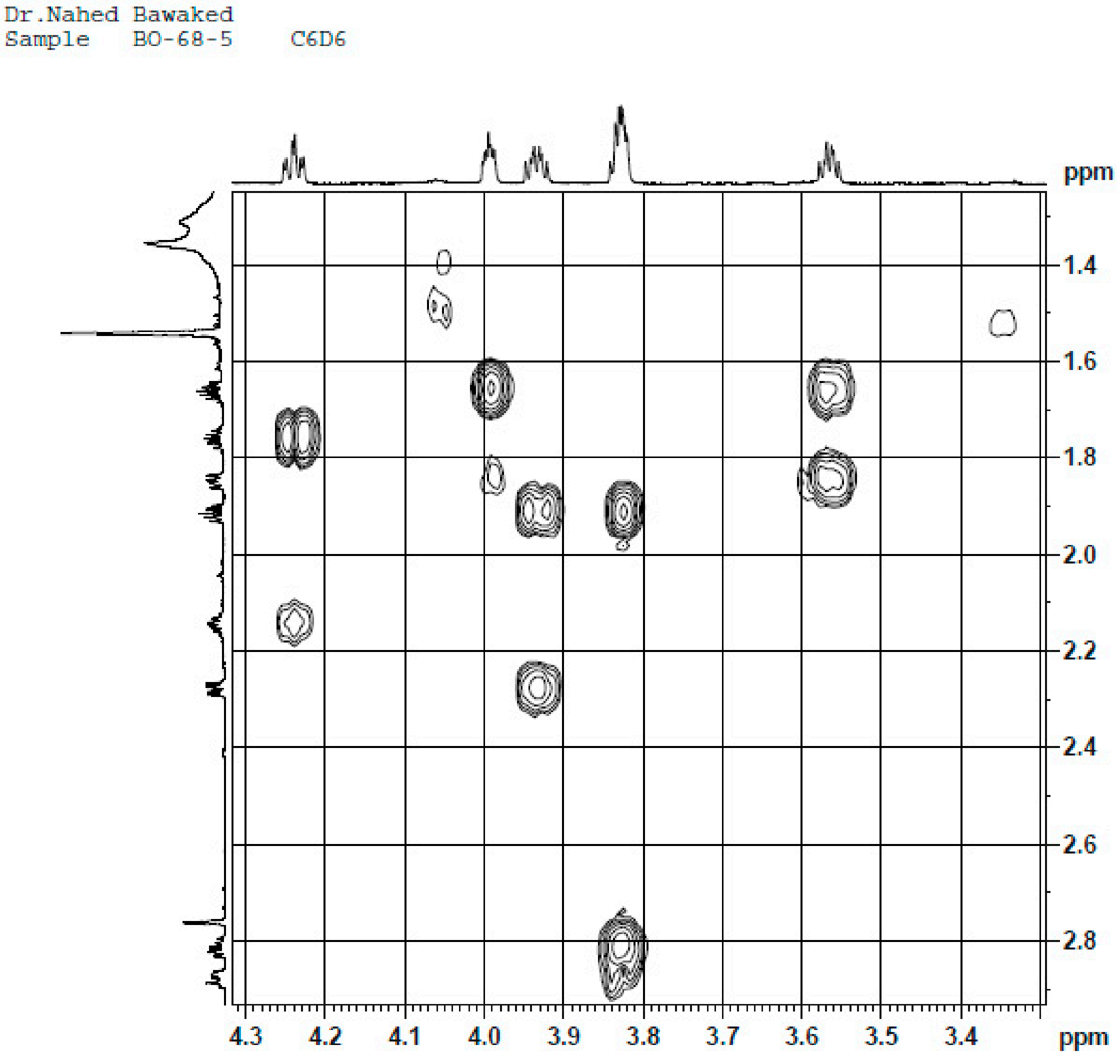

Figure S2k: COSY NMR of compound 2

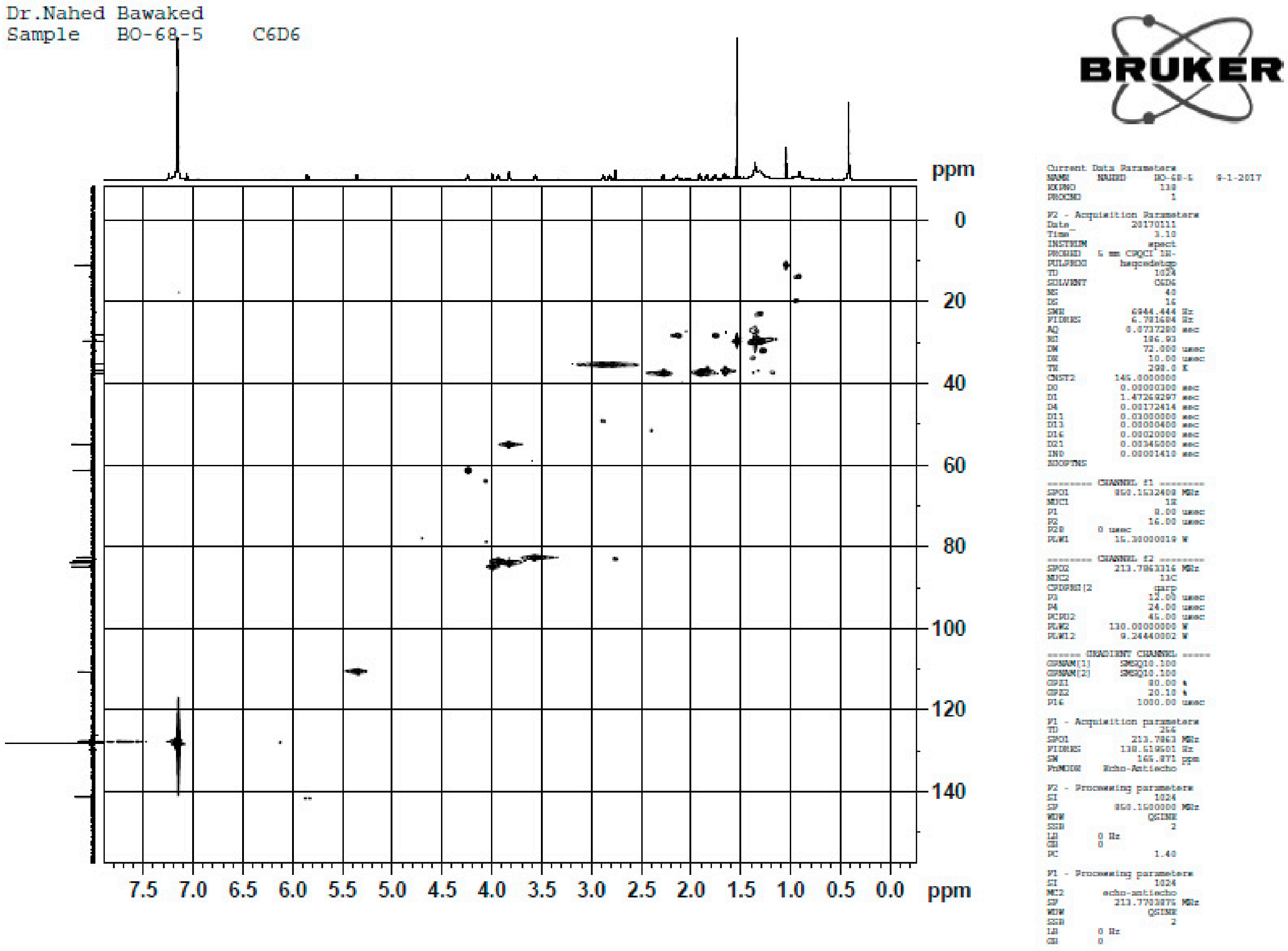

Figure S2l: HSQC NMR of compound 2

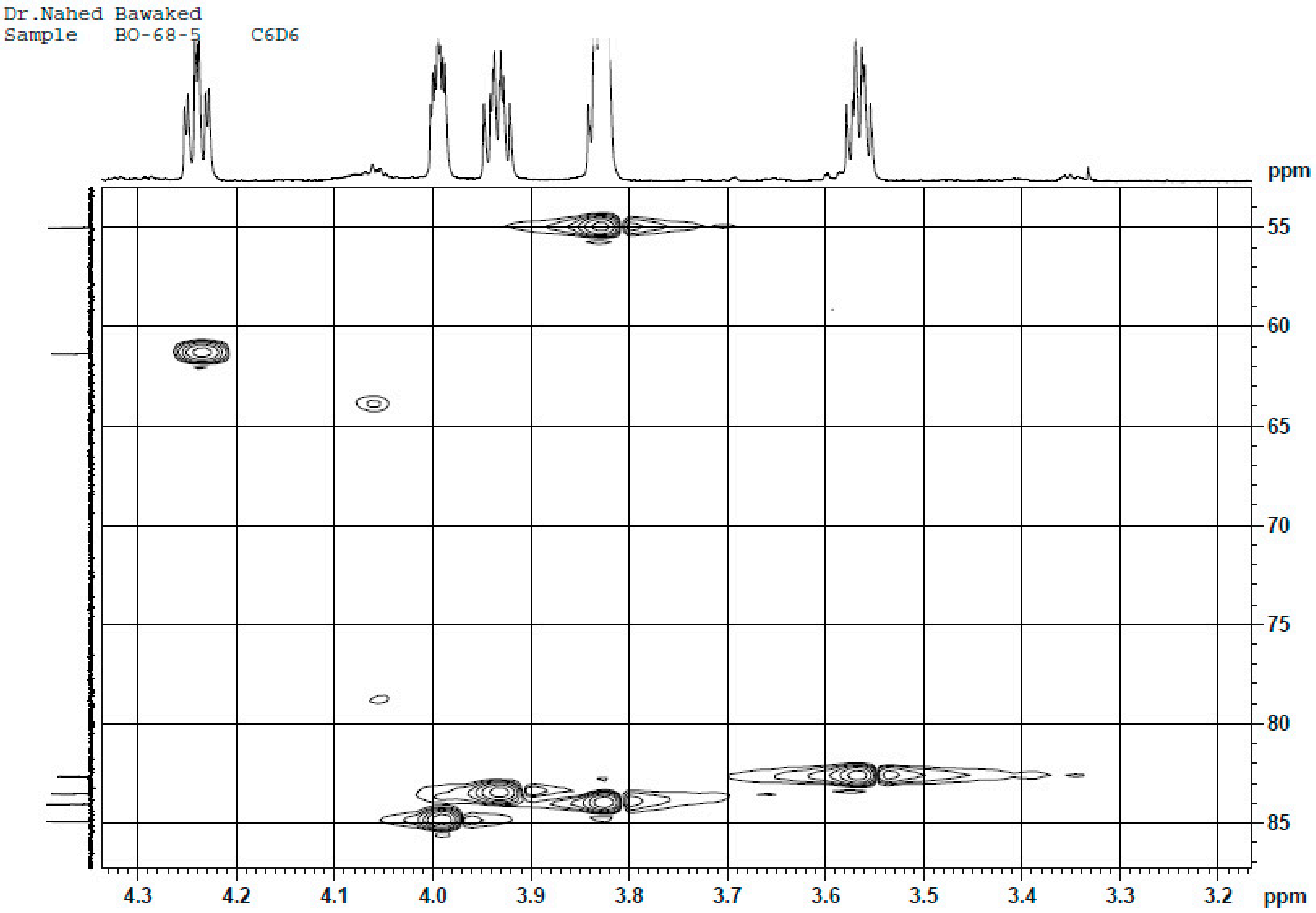

Figure S2m: HSQC NMR of compound 2

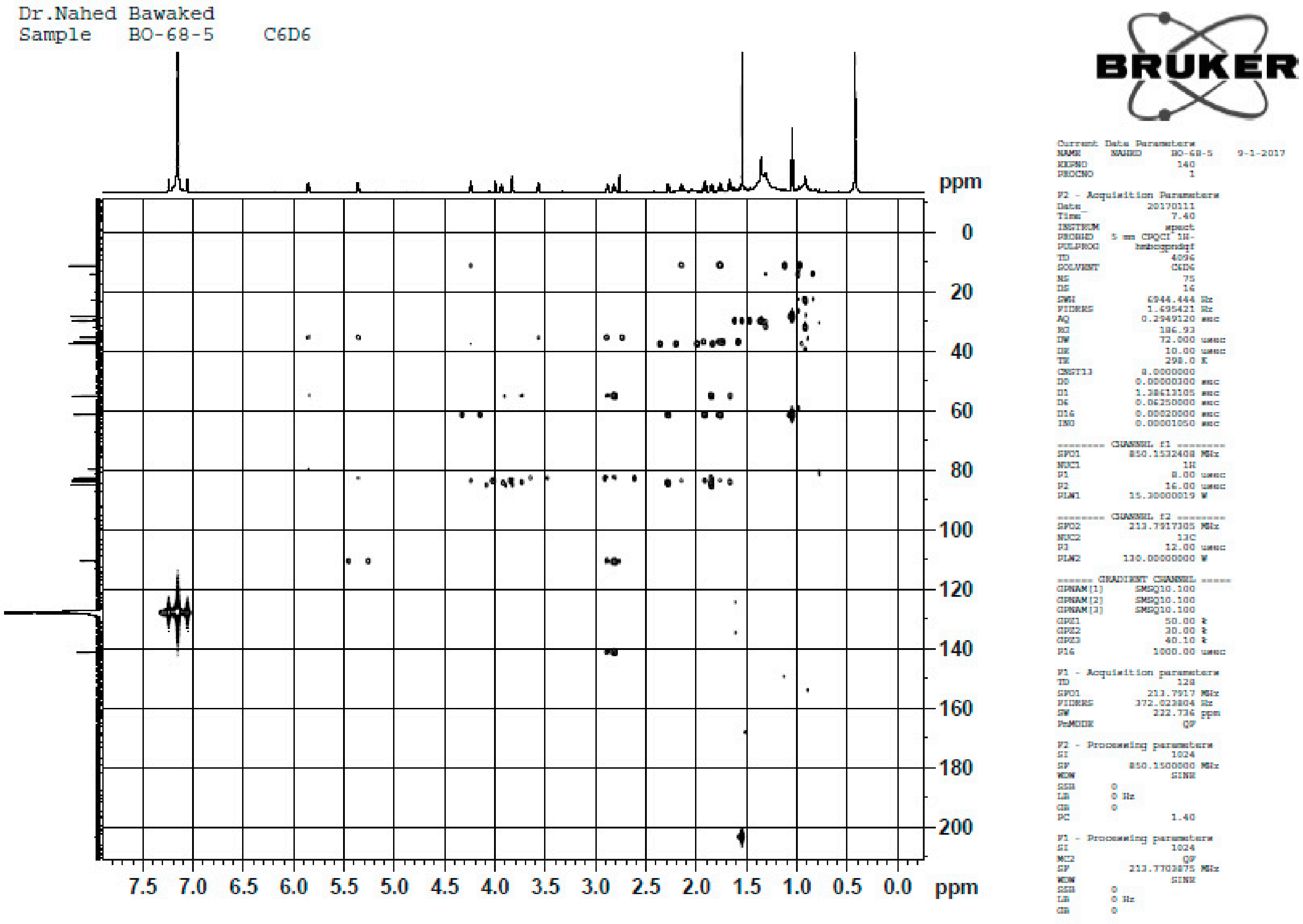

Figure S2n: HMBC NMR of compound 2

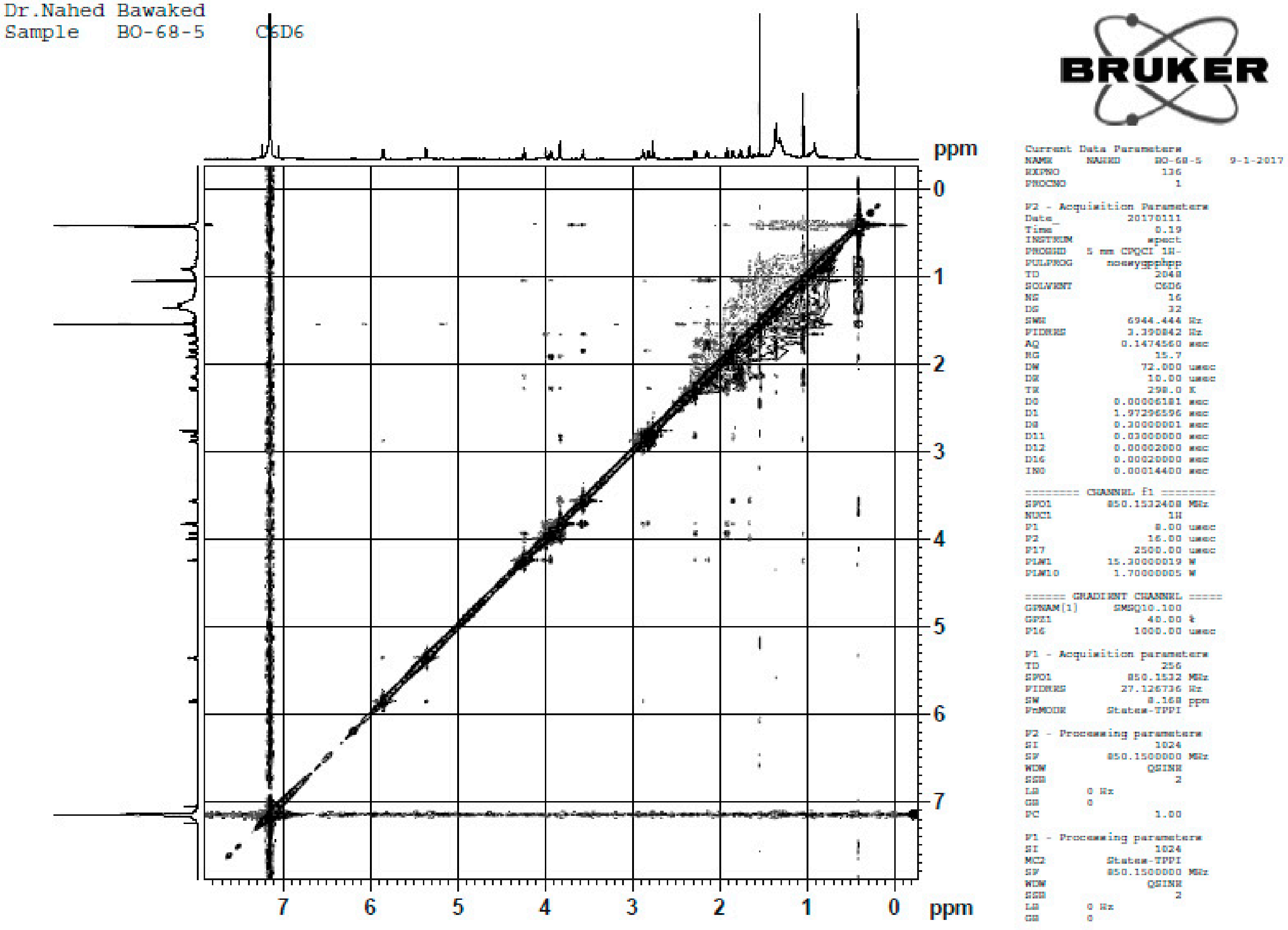

Figure S2o: NOESY NMR of compound 2

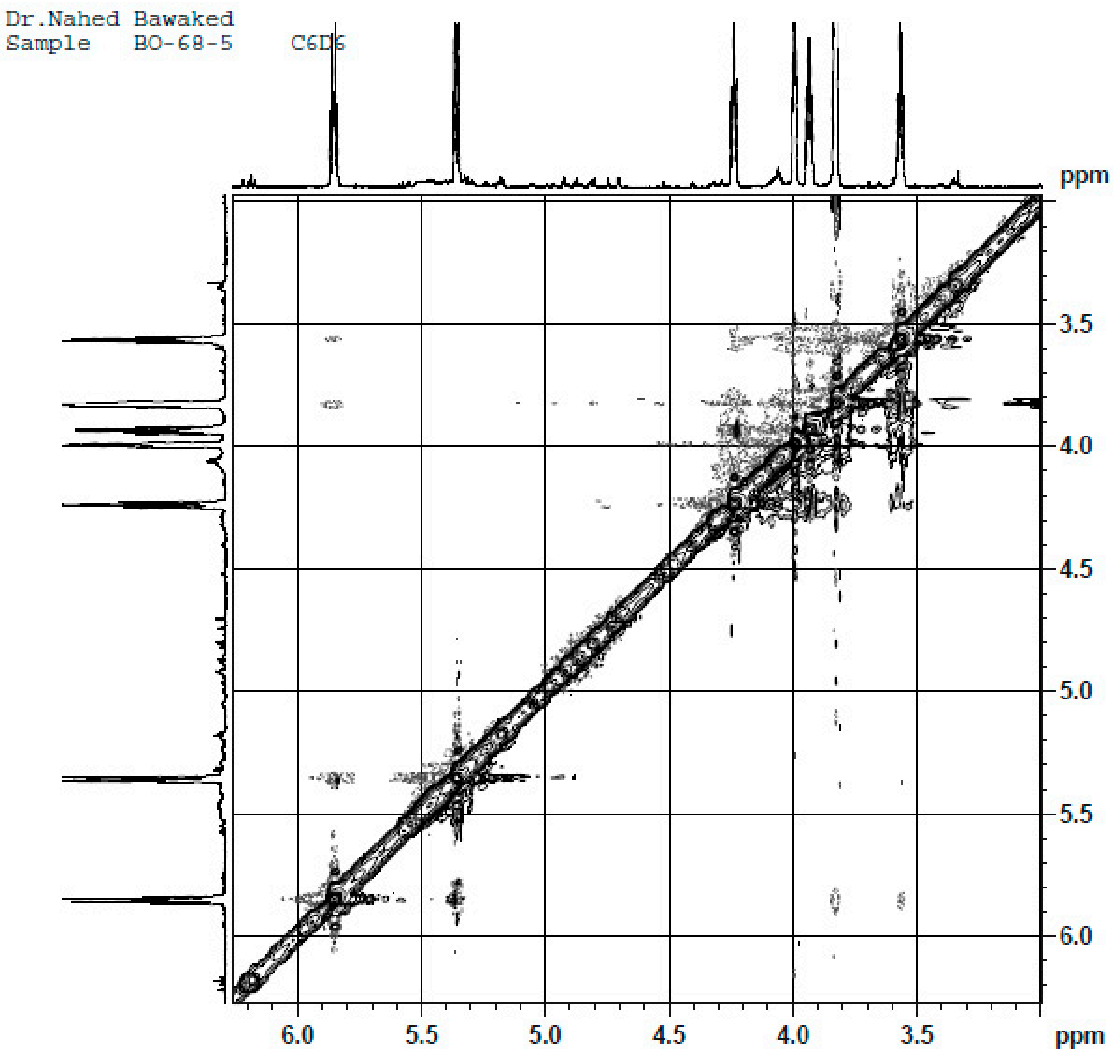

Figure S2p: NOESY NMR of compound 2
